# Supplementary material for: METTL14-mediated Lnc-LSG1 m6A modification inhibits clear cell renal cell carcinoma metastasis via regulating ESRP2 ubiquitination
Source: Mol Ther Nucleic Acids. 2021 Dec 17;27:547–61. doi: 10.1016/j.omtn.2021.12.024 (PMC8738955; doi:10.1016/j.omtn.2021.12.024)
Supplement: Document S1. Figures S1–S4 and Table S1–S6 [file mmc1.pdf]

OMTN, Volume *27*

Supplemental information

METTL14-mediated Lnc-LSG1 m6A modification inhibits clear cell renal cell carcinoma metastasis via regulating ESRP2 ubiquitination

Danyang Shen, Lifeng Ding, Zeyi Lu, Ruyue Wang, Chenhao Yu, Huan Wang, Qiming Zheng, Xuliang Wang, Wanjiang Xu, Haifeng Yu, Liwei Xu, Mingchao Wang, Shicheng Yu, Shibin Zhu, Jun Qian, Liqun Xia, and Gonghui Li

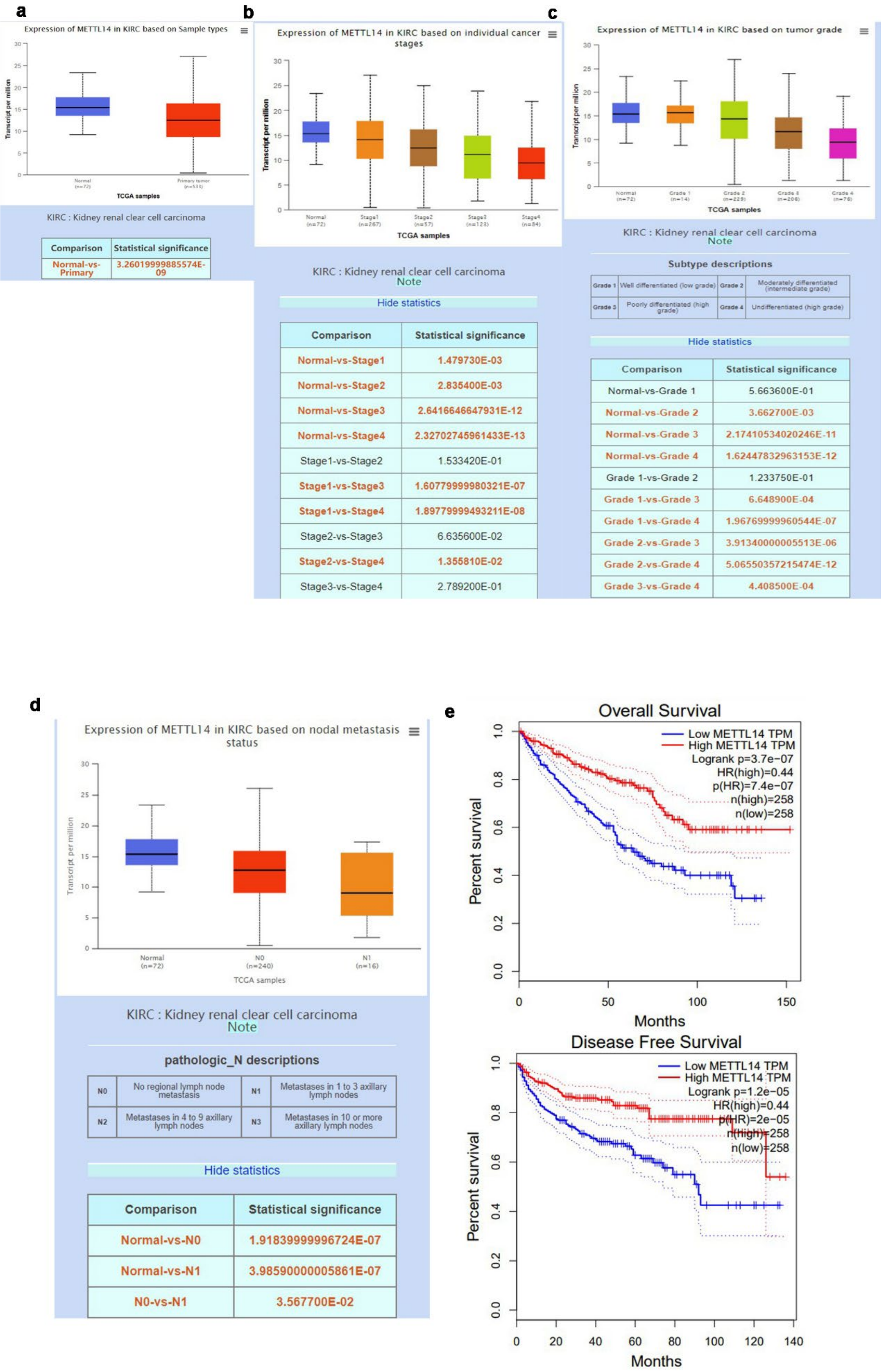
a
Expression of METTL14 in KIRC based on Sample types
Transcript per million
TCGA samples
KIRC : Kidney renal clear cell carcinoma
Comparison | Statistical significance
Normal-vs-Primary | 3.26019999885574E-09
b
Expression of METTL14 in KIRC based on individual cancer stages
Transcript per million
Normal (n=72)
Stage1 (n=267)
Stage2 (n=57)
Stage3 (n=123)
Stage4 (n=84)
TCGA samples
KIRC : Kidney renal clear cell carcinoma
Note
Hide statistics
Comparison | Statistical significance
Normal-vs-Stage1 | 1.479730E-03
Normal-vs-Stage2 | 2.835400E-03
Normal-vs-Stage3 | 2.6416646647931E-12
Normal-vs-Stage4 | 2.32702745961433E-13
Stage1-vs-Stage2 | 1.533420E-01
Stage1-vs-Stage3 | 1.60779999980321E-07
Stage1-vs-Stage4 | 1.89779999493211E-08
Stage2-vs-Stage3 | 6.635600E-02
Stage2-vs-Stage4 | 1.355810E-02
Stage3-vs-Stage4 | 2.789200E-01
c
Expression of METTL14 in KIRC based on tumor grade
Transcript per million
TCGA samples
KIRC : Kidney renal clear cell carcinoma
Note
Subtype descriptions
Grade 1 | Well differentiated (low grade) | Grade 2 | Moderately differentiated (intermediate grade)
Grade 3 | Poorly differentiated (high grade) | Grade 4 | Undifferentiated (high grade)
Hide statistics
Comparison | Statistical significance
Normal-vs-Grade 1 | 5.663600E-01
Normal-vs-Grade 2 | 3.662700E-03
Normal-vs-Grade 3 | 2.17410534020246E-11
Normal-vs-Grade 4 | 1.62447832963153E-12
Grade 1-vs-Grade 2 | 1.233750E-01
Grade 1-vs-Grade 3 | 6.648900E-04
Grade 1-vs-Grade 4 | 1.96769999960544E-07
Grade 2-vs-Grade 3 | 3.91340000005513E-06
Grade 2-vs-Grade 4 | 5.06550357215474E-12
Grade 3-vs-Grade 4 | 4.408500E-04
d
Expression of METTL14 in KIRC based on nodal metastasis status
Transcript per million
Normal (n=72)
N0 (n=240)
N1 (n=16)
TCGA samples
KIRC : Kidney renal clear cell carcinoma
Note
pathologic_N descriptions
N0 | No regional lymph node metastasis | N1 | Metastases in 1 to 3 axillary lymph nodes
N2 | Metastases in 4 to 9 axillary lymph nodes | N3 | Metastases in 10 or more axillary lymph nodes
Hide statistics
Comparison | Statistical significance
Normal-vs-N0 | 1.91839999996724E-07
Normal-vs-N1 | 3.98590000005861E-07
N0-vs-N1 | 3.567700E-02
e
Overall Survival
Low METTL14 TPM
High METTL14 TPM
Logrank p=3.7e−07
HR(high)=0.44
p(HR)=7.4e−07
n(high)=258
n(low)=258
Percent survival
Months
Disease Free Survival
Low METTL14 TPM
High METTL14 TPM
Logrank p=1.2e−05
HR(high)=0.44
p(HR)=2e−05
n(high)=258
n(low)=258
Percent survival
Months

Figure S1. (a), (b), (c). According to the UAlCAN database, METTL14 was downregulated in the ccRCC primary tumor when compared to normal tissues (a). The higher the tumor stage (b) and grade (c), the lower the METTL14 expression. (d). METTL14 expression was lower in metastatic ccRCC tissues than that in non-metastatic tissues. (d). Kaplan-Meier analysis in the GEPIA database showed that lower METTL14 expression is associated with poorer OS and DFS. All these data were obtained on 03/16/2021. Data were presented as the mean ± SD of at least three independent experiments.

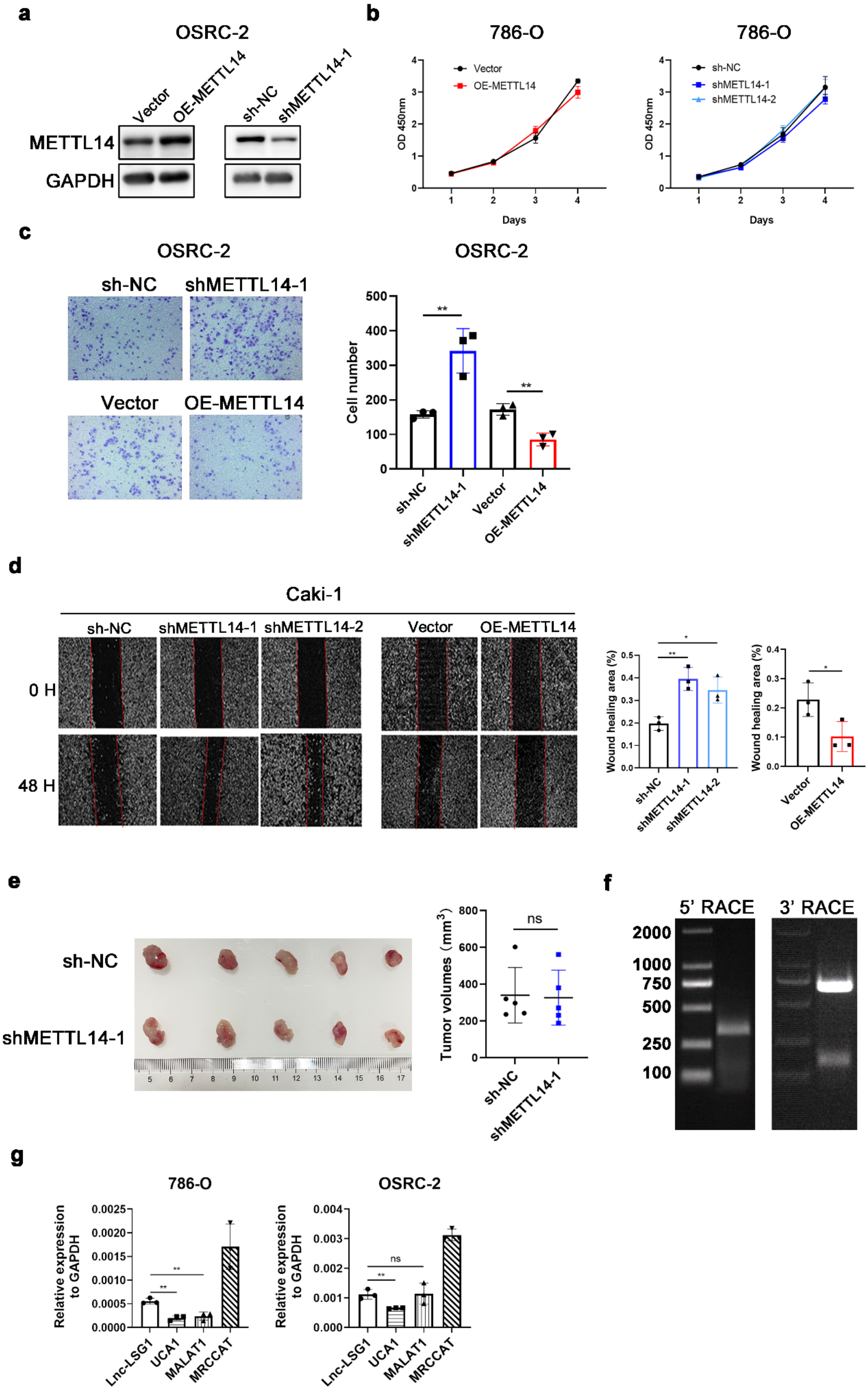
a
OSRC-2
Vector
OE-METTL14
sh-NC
shMETTL14-1
METTL14
GAPDH
b
786-O
Vector
OE-METTL14
sh-NC
shMETTL14-1
shMETTL14-2
OD 450nm
Days
c
OSRC-2
sh-NC
shMETTL14-1
Vector
OE-METTL14
Cell number
d
Caki-1
sh-NC
shMETTL14-1
shMETTL14-2
Vector
OE-METTL14
0 H
48 H
Wound healing area (%)
e
sh-NC
shMETTL14-1
Tumor volumes（mm³）
ns
f
5' RACE
3' RACE
2000
1000
750
500
250
100
g
786-O
OSRC-2
Relative expression to GAPDH
Lnc-LSG1
UCA1
MALAT1
MRCCAT
ns

h

ID	Label	Coding probability	Peptide length(aa)	Fickett score	Isoelectric point	ORF integrity
Lnc-LSG1	noncoding	0.0318156	64	0.3284	8.90765380859	complete

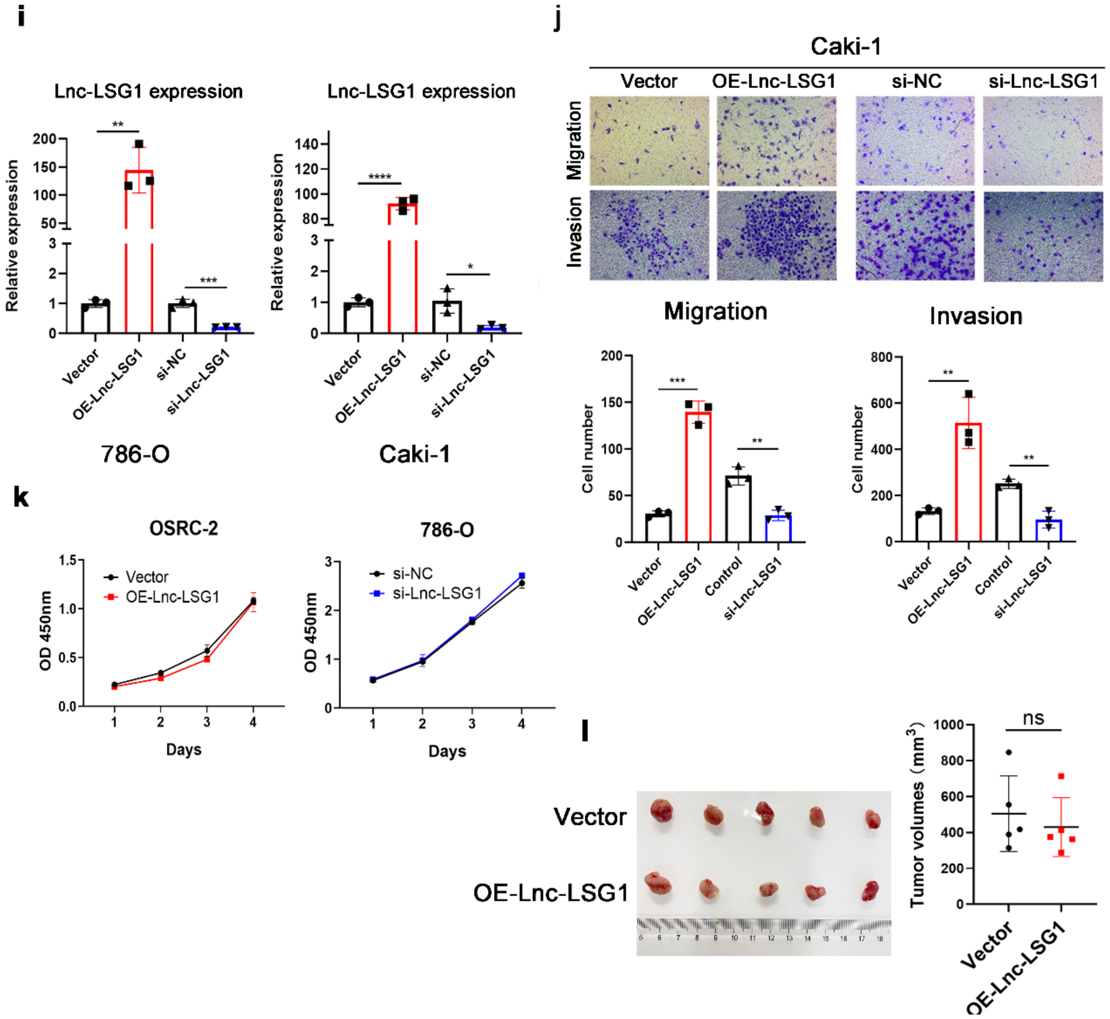

Figure S2 (a). METTL14 knockdown and overexpression were confirmed by western blot assay in OSRC-2 cells. (b). METTL14 cannot regulate 786-O proliferation. (c). Transwell assay showed that METTL14 inhibits OSRC-2 migration. (d). Wound healing assay were performed to detect the migratory and invasive abilities of Caki-1 cells after METTL14 depletion and overexpression. Magnification, 100×. (e). The mice were sacrificed and tumor volume was measured after 4 weeks OSRC-2 cells injection. Left panel: Image of the xenograft tumors from sh-NC and sh-METTL14-1 group. Right panel: Average tumor volume with SD (n = 5 per group). (f). 5′ and 3′

RACE assays in 786-O cells detected the whole sequence of Lnc-LSG1.The figure showed PCR products from the 5′-RACE and 3′-RACE assays. (g). The expression of Lnc-LSG1 and some highly expressed lncRNAs in ccRCC cells. (h). Protein coding potential of Lnc-LSG1-4:2 as predicted by CPC 2.0. (i). Lnc-LSG1 knockdown and overexpression were confirmed by RT-qPCR assay. (j). Transwell assay showed the regulatory effect of Lnc-LSG1 on Caki-1 migration and invasion. (k). CCK8 assay showed Lnc-LSG1 has no effect on cell proliferation. (l). The mice were sacrificed and tumor volume was measured after 4 weeks OSRC-2 cells injection. Left panel: Image of the xenograft tumors from Vector and OE-Lnc-LSG1 group. Right panel: Average tumor volume with SD (n = 5 per group). *P < 0.05, **P < 0.01, ***P < 0.001, ****P<0.0001. ns: no significance. Data were presented as the mean ± SD of at least three independent experiments.

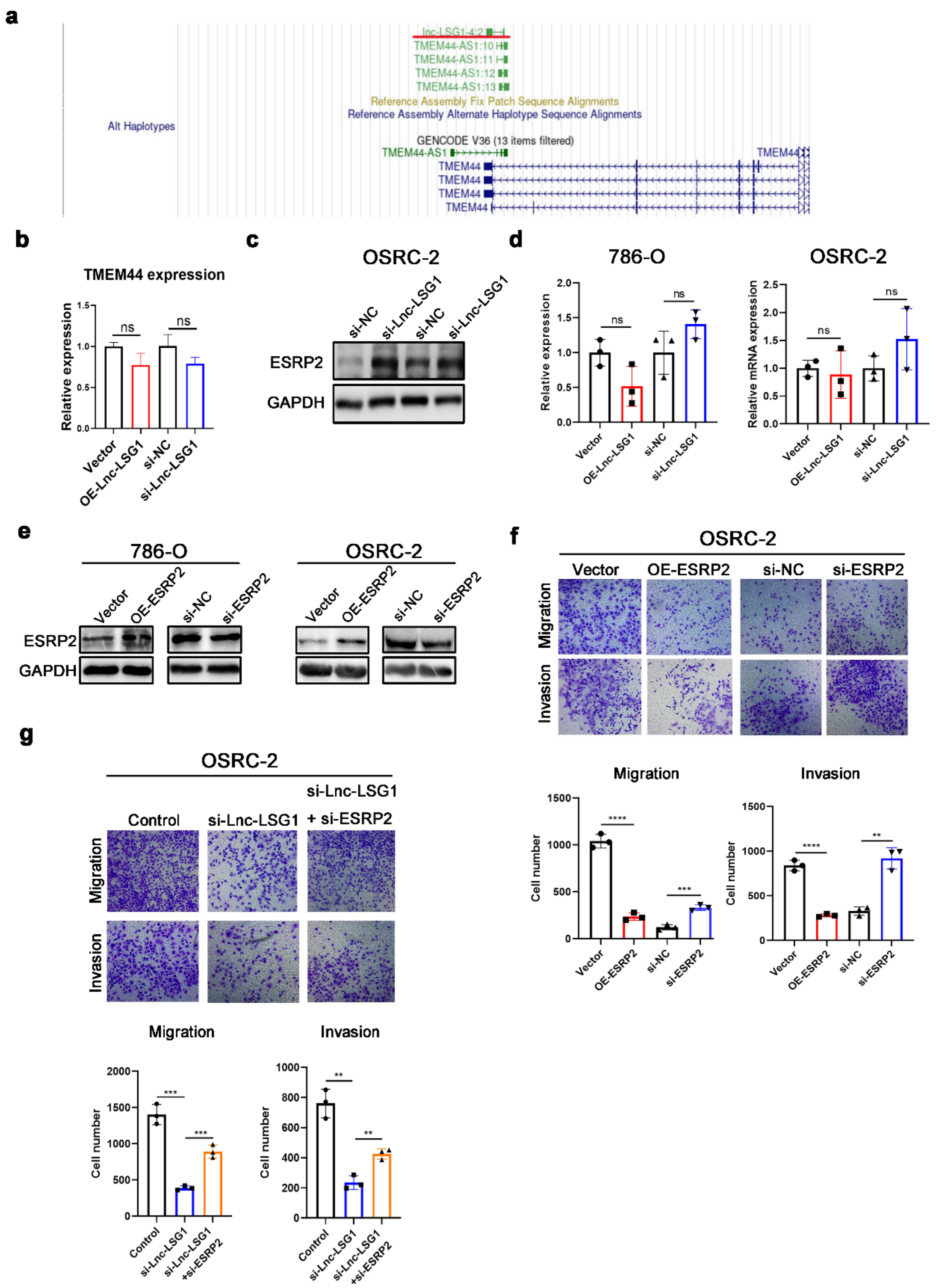

Figure S3. (a). The location of Lnc-LSG1-4:2 was retrieved by the UCSC (http://genome.ucsc.edu/) database. These data were obtained on 03/16/2021. (b). Lnc-LSG1 showed no effect on TMEM44 mRNA expression. (c). si-Lnc-LSG1 can increase ESPR2 protein level. (d). Lnc-LSG1 showed no effect on ESRP2 mRNA

expression. (e). Knockdown and overexpression ESRP2 were confirmed by western blot assay. (f). Transwell assay showed that ESRP2 can inhibit the migration and invasion of OSRC-2 cells. (g). si-ESRP2 can rescue the inhibited migratory and invasive ability of OSRC-2 cells induced by si-Lnc-LSG1. ns: no significance. *P < 0.05, **P < 0.01, ***P < 0.001, ****P<0.0001, ns: no significance. Data were presented as the mean ± SD of at least three independent experiments.

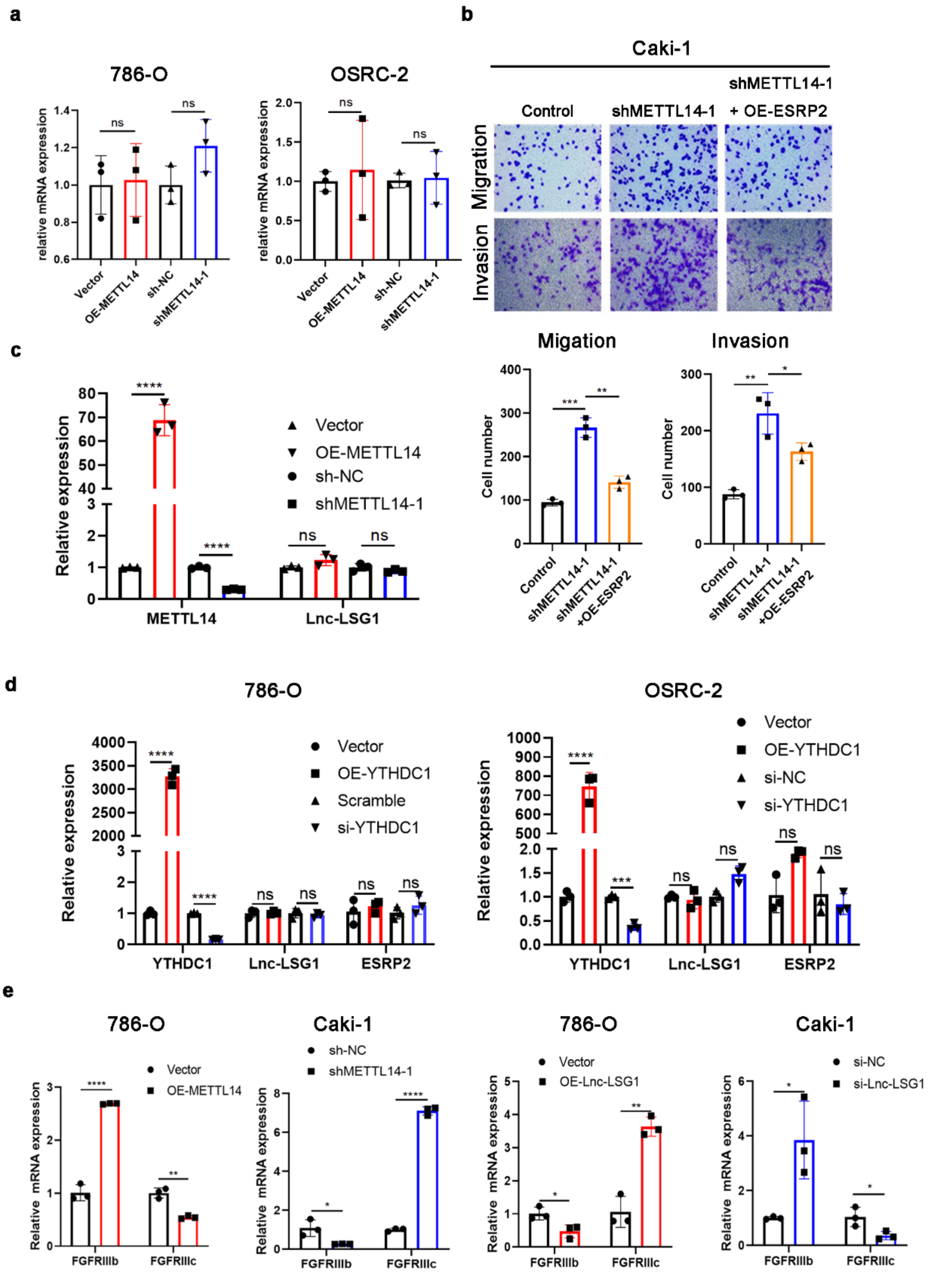

Figure S4 (a). RT-qPCR assay showed that METTL14 cannot regulate ESRP2 mRNA expression. (b). ESRP2 overexpression can reverse the enhanced migration and invasion of Caki-1 cells induced by shMETTL14-1. (c). METTL14 cannot regulate Lnc-LSG1 expression. (d). YTHDC1 cannot regulate Lnc-LSG1 and ESRP2 mRNA

expression. (e). RT-qPCR assay revealed the regulatory effect of METTL14 and Lnc-LSG1 on FGFR2 IIIb and FGFR2IIIc. *$P < 0.05$, **$P < 0.01$, ***$P < 0.001$, ****$P<0.0001$, ns: no significance. Data were presented as the mean ± SD of at least three independent experiments.

Table S1. Information of patients from Sir Run Run Shaw Hospital （RT-qPCR samples）									
NO	Gender	Age	Metastasis	Pathology	Stage	Grade	T	N	M
1	Male	48	no	ccRCC	I	2	1b	0	0
2	Male	56	no	ccRCC	III	3	3a	0	0
3	Male	84	no	ccRCC	I	2	1b	0	0
4	Male	54	no	ccRCC	II	3	2a	0	0
5	Male	81	no	ccRCC	I	3	1a	0	0
6	Male	76	no	ccRCC	I	3	1a	0	0
7	Male	50	no	ccRCC	I	2	1a	0	0
8	Male	71	no	ccRCC	I	3	1b	0	0
9	Male	59	no	ccRCC	I	2	1a	0	0
10	Male	55	no	ccRCC	II	3	2a	0	0
11	Female	75	no	ccRCC	I	2	1b	0	0
12	Male	72	no	ccRCC	I	3	1a	0	0
13	Female	73	no	ccRCC	I	2	1b	0	0
14	Male	60	no	ccRCC	I	3	1a	0	0
15	Male	37	no	ccRCC	I	2	1a	0	0
16	Male	70	no	ccRCC	I	3	1a	0	0
17	Male	41	no	ccRCC	II	2	2b	0	0
18	Male	57	no	ccRCC	I	3	1a	0	0
19	Male	60	no	ccRCC	I	3	1a	0	0
20	Female	45	no	ccRCC	II	3	2a	0	0
21	Male	42	no	ccRCC	I	3	1a	0	0
22	Male	54	no	ccRCC	I	2	1b	0	0
23	Male	49	no	ccRCC	I	2	1a	0	0
24	Female	56	no	ccRCC	II	2	2a	0	0
25	Female	70	no	ccRCC	I	2	1b	0	0
26	Female	61	no	ccRCC	I	2	1b	0	0
27	Male	69	no	ccRCC	I	2	1b	0	0
28	Male	32	no	ccRCC	I	2	1a	0	0
29	Male	81	no	ccRCC	I	2	1b	0	0
30	Female	52	no	ccRCC	I	2	1a	0	0
31	Male	79	no	ccRCC	I	3	1a	0	0
32	Male	31	no	ccRCC	I	2	1b	0	0
33	Female	85	no	ccRCC	I	2	1b	0	0
34	Male	56	no	ccRCC	I	2	1a	0	0
35	Male	46	no	ccRCC	I	2	1a	0	0
36	Female	55	no	ccRCC	I	2	1a	0	0
37	Male	70	no	ccRCC	I	2	1a	0	0
38	Female	79	no	ccRCC	I	2	1b	0	0
39	Male	63	no	ccRCC	I	2	1b	0	0
40	Male	56	no	ccRCC	I	2	1a	0	0
41	Male	37	no	ccRCC	I	2	1b	0	0
42	Male	72	no	ccRCC	I	2	1b	0	0
43	Male	40	no	ccRCC	I	2	1a	0	0
44	Male	64	no	ccRCC	II	2	2a	0	0
45	Female	52	no	ccRCC	I	2	1a	0	0
46	Male	58	no	ccRCC	I	2	1a	0	0
47	Male	57	no	ccRCC	I	2	1a	0	0
48	Male	60	no	ccRCC	I	3	1a	0	0
49	Female	73	no	ccRCC	I	2	1b	0	0
50	Female	51	no	ccRCC	I	2	1a	0	0

Table S2. Information of patients from Sir Run Run Shaw Hospital （IHC samples）										
NO	Gender	Age	Metastasis	Pathology	Stage	Grade	Tumor Size (cm)	T	N	M
1	M	60	no	ccRCC	II	I	8*5	T2a	N0	M0
2	F	52	no	ccRCC	I	IV	4.8*4.5	T1b	N0	M0
3	M	71	no	ccRCC	I	II	4*3*3	T1a	N0	M0
4	F	51	no	ccRCC	I	II	3*2.5*1.7	T1a	N0	M0
5	M	42	no	ccRCC	II	III	8.3*8*5.5	T2a	N0	M0
6	M	60	no	ccRCC	I	II	4*3.7	T1a	N0	M0
7	F	60	no	ccRCC	I	II	3.5*3.5	T1a	N0	M0
8	M	62	no	ccRCC	I	III	2.3*2.2	T1a	N0	M0
9	F	70	no	ccRCC	I	III	3.8*3.5	T1a	N0	M0
10	M	43	no	ccRCC	I	II	7*4.7	T1b	N0	M0
11	M	60	no	ccRCC	I	III	3*3	T1a	N0	M0
12	M	33	no	ccRCC	I	IV	5.7*4	T1b	N0	M0
13	M	52	no	ccRCC	I	II	1.8*1.6	T1a	N0	M0
14	M	51	no	ccRCC	I	II	3.5*3	T1a	N0	M0
15	F	50	no	ccRCC	I	I	1.2*1	T1a	N0	M0
16	M	59	no	ccRCC	I	II	2.2*1.8	T1a	N0	M0
17	M	79	no	ccRCC	I	III	4.5*4	T1b	N0	M0
18	M	50	no	ccRCC	I	III	5.1*3.4	T1b	N0	M0
19	F	67	no	ccRCC	I	II	2.2*1.6	T1a	N0	M0
20	F	73	no	ccRCC	I	III	7*5.8	T1b	N0	M0
21	M	43	no	ccRCC	I	I	3.7*3.5	T1a	N0	M0
22	M	44	no	ccRCC	I	II	3.8*3.5	T1a	N0	M0
23	M	40	no	ccRCC	I	III	2.6*2.2*1.7	T1a	N0	M0
24	F	55	no	ccRCC	I	I	1.2*1.2	T1a	N0	M0
25	F	28	no	ccRCC	I	II	6.2*5.8	T1b	N0	M0
26	M	56	no	ccRCC	I	II	6.3*5.1	T1b	N0	M0
27	M	60	no	ccRCC	I	II	2.7*2.4	T1a	N0	M0
28	M	70	no	ccRCC	I	III	1.7*1.6	T1a	N0	M0
29	F	57	no	ccRCC	I	II	6*4.5*3.5	T1b	N0	M0
30	M	54	no	ccRCC	I	II	5.3*3	T1b	N0	M0
31	M	34	no	ccRCC	I	I	1.2*1.2	T1a	N0	M0
32	F	74	no	ccRCC	I	II	5*3.2	T1b	N0	M0
33	M	43	no	ccRCC	I	II	2.6*1.8	T1a	N0	M0
34	M	45	no	ccRCC	I	II	1.8*1.8	T1a	N0	M0
35	M	64	no	ccRCC	I	II	3.2*2.4	T1a	N0	M0
36	F	46	no	ccRCC	II	II	7.2*6.5	T2a	N0	M0
37	F	51	no	ccRCC	I	II	3.3*3	T1a	N0	M0
38	M	54	no	ccRCC	I	II	2.5*2	T1a	N0	M0
39	M	66	no	ccRCC	II	IV	9*7.5	T2a	N0	M0
40	F	71	no	ccRCC	I	II	4.5*4	T1b	N0	M0

Table S3. Information of patients from tissue microarray

NO	Gender	Age	Metastasis	Pathology	Stage	Grade	Tumor Size (cm)	T	N	M	Survival time (month)	Status
1	F	52	no	ccRCC	I	II	6.5×6×5	T1b	N0	M0	15.4	Dead
2	M	56	no	ccRCC	III	I	3.5×3×3	T3a	N0	M0	97.8	Alive
3	F	73	no	ccRCC	I	I	5×5×4	T1b	N0	M0	59.20	Alive
4	M	55	no	ccRCC	I	II	5×4×4	T1b	N0	M0	97.34	Alive
5	M	59	no	ccRCC	I	II	5.5×5×5	T1b	N0	M0	97.34	Alive
6	M	69	no	ccRCC	I	II	4.5×4×4	T1b	N0	M0	97.11	Alive
7	M	72	no	ccRCC	II	I	10×6×5	T2a	N0	M0	93.9	Alive
8	M	59	no	ccRCC	II	II	15×9×6.5	T2b	N0	M0	7.2	Dead
9	M	63	no	ccRCC	I	II	7×5×3.5	T1b	N0	M0	95.93	Alive
10	F	52	no	ccRCC	I	II	7×7×6	T1b	N0	M0	95.64	Alive
11	M	67	no	ccRCC	I	I	2.8×2.5×2.5	T1a	N0	M0	95.51	Alive
12	M	66	no	ccRCC	II	II	8×8×7	T2a	N0	M0	95.41	Alive
13	F	57	no	ccRCC	II	II	8×7×5.5	T2a	N0	M0	56.1	Dead
14	M	57	no	ccRCC	III	IV	12×8.5×7	T3b	N0	M0	5.6	Dead
15	M	61	no	ccRCC	I	II	7×5×5	T1b	N0	M0	95.02	Alive
16	F	79	no	ccRCC	I	II	3×3×2.5	T1a	N0	M0	22.5	Dead
17	F	49	no	ccRCC	I	I	1.2×1×0.8	T1a	N0	M0	94.03	Alive
18	F	72	no	ccRCC	I	II	6×5.5×5	T1b	N0	M0	93.38	Alive
19	F	64	no	ccRCC	I	II	3×3×3	T1a	N0	M0	92.75	Alive
20	M	38	no	ccRCC	I	I	2.5×2×2	T1a	N0	M0	92.69	Alive
21	M	58	no	ccRCC	I	II	4.5×4×3	T1b	N0	M0	92.00	Alive
22	F	61	no	ccRCC	I	II	5×4.5×4	T1b	N0	M0	91.93	Alive
23	M	60	no	ccRCC	II	III	9×8×6	T2a	N0	M0	91.57	Alive
24	M	55	no	ccRCC	I	III	4×3.5×3.5	T1a	N0	M0	91.51	Alive
25	M	75	no	ccRCC	III	III	4×3.5×3	T3a	N0	M0	91.5	Alive
26	F	63	no	ccRCC	I	II	3.5×2.5×2	T1a	N0	M0	91.15	Alive
27	F	45	no	ccRCC	II	II	8×7×6	T2a	N0	M0	90.92	Alive
28	M	51	no	ccRCC	I	II	2×2×1.5	T1a	N0	M0	90.79	Alive
29	M	40	no	ccRCC	I	II	6×3.5×3.5	T1b	N0	M0	90.13	Alive
30	M	78	no	ccRCC	I	III	5×5×4.5	T1b	N0	M0	90.00	Alive
31	M	53	no	ccRCC	I	I	7×6×5	T1b	N0	M0	89.93	Alive
32	F	61	no	ccRCC	I	II	5×4×4	T1b	N0	M0	89.41	Alive
33	M	61	no	ccRCC	III	II	8×8×6	T3a	N0	M0	88.6	Alive
34	M	62	no	ccRCC	I	II	7×6×5	T1b	N0	M0	88.52	Alive
35	M	63	no	ccRCC	I	II	7×6×4	T1b	N0	M0	98.9	Alive
36	F	60	no	ccRCC	I	II	6×6×5	T1b	N0	M0	87.21	Alive
37	M	64	no	ccRCC	III	III	7×6×6	T3b	N1	M0	7.8	Dead
38	M	60	no	ccRCC	II	III	8×7×6	T2a	N0	M0	43.9	Dead
39	M	60	no	ccRCC	I	II	4×3.5×3.5	T1a	N0	M0	86.92	Alive
40	F	33	no	ccRCC	I	II	2.5×2.5×2	T1a	N0	M0	86.69	Alive
41	M	67	no	ccRCC	III	II	7×5.5×4	T3a	N0	M0	59.4	Dead
42	M	55	no	ccRCC	I	I	5×4×4	T1b	N0	M0	86.33	Alive
43	M	52	no	ccRCC	I	I	6.5×5.5×5.5	T1b	N0	M0	86.33	Alive
44	M	57	no	ccRCC	I	I	6×5.5×	T1b	N0	M0	86.33	Alive
45	M	52	no	ccRCC	I	I	5×4×3.5	T1b	N0	M0	86.30	Alive
46	M	60	no	ccRCC	III	IV	7×7×6	T3a	N0	M0	8.6	Dead
47	M	64	no	ccRCC	II	II	10×10×7	T2a	N0	M0	20.6	Dead
48	M	65	no	ccRCC	III	IV	5.5×5×4.5	T3a	N0	M0	9.5	Dead
49	F	75	no	ccRCC	I	I	5×4.5×4	T1b	N0	M0	84.69	Alive
50	F	61	no	ccRCC	III	IV	12×10×7	T3a	N0	M0	45.2	Dead
51	M	69	no	ccRCC	I	III	5×4.5×4.5	T1b	N0	M0	84.39	Alive
52	M	73	no	ccRCC	I	II	2.5×2.5×2	T1a	N0	M0	83.97	Alive
53	F	68	no	ccRCC	I	II	4.5×4.5×4	T1b	N0	M0	83.74	Alive
54	M	71	no	ccRCC	I	II	5×5×4.5	T1b	N0	M0	83.74	Alive
55	F	65	no	ccRCC	I	I	5×3.5×4	T1b	N0	M0	83.34	Alive
56	M	86	no	ccRCC	II	III	9.5×7×5.5	T2a	N0	M0	33.80	Dead
57	M	65	no	ccRCC	II	III	10×6×6	T2a	N0	M0	37.2	Dead
58	M	61	no	ccRCC	I	I	3.5×3×2.5	T1a	N0	M0	82.62	Alive
59	F	72	no	ccRCC	I	II	5×4.5×4	T1b	N0	M0	82.07	Alive
60	M	58	no	ccRCC	I	I	4.5×4×3.8	T1b	N0	M0	82.03	Alive
61	M	68	no	ccRCC	III	IV	6.5×6.5×5.5	T3a	N0	M0	11.3	Dead
62	M	74	no	ccRCC	II	III	8×8×5	T2a	N0	M0	81.25	Alive
63	M	38	no	ccRCC	III	IV	5.5×4.5×4	T3a	N0	M0	12.6	Dead
64	M	33	no	ccRCC	I	III	7×6×5	T1b	N0	M0	80.79	Alive
65	M	75	no	ccRCC	I	II	4×3×2.5	T1a	N0	M0	80.59	Alive
66	F	64	no	ccRCC	I	II	6.5×5.5×5	T1b	N0	M0	80.56	Alive
67	F	59	no	ccRCC	I	I	3×3×2.5	T1a	N0	M0	80.26	Alive
68	M	59	no	ccRCC	I	II	3.5×3.5×2.5	T1a	N0	M0	80.13	Alive
69	M	58	no	ccRCC	I	I	4.5×4.5×4	T1b	N0	M0	79.67	Alive
70	M	55	no	ccRCC	I	II	4×3×2.5	T1a	N0	M0	79.64	Alive
71	F	81	no	ccRCC	I	II	6×5.5×4.5	T1b	N0	M0	6.6	Dead
72	F	59	no	ccRCC	I	I	5.5×5×5	T1b	N0	M0	79.44	Alive
73	M	60	no	ccRCC	III	III	7×5×5	T3a	N0	M0	15.7	Dead
74	M	67	no	ccRCC	I	II	6×5×5	T1b	N0	M0	78.30	Alive
75	M	34	no	ccRCC	III	II	13×6.5×5.5	T3a	N0	M0	3.9	Dead
76	M	48	no	ccRCC	III	III	5.5×4.5×4	T3a	N0	M0	15.2	Dead
77	M	63	no	ccRCC	I	III	5×4×2	T1b	N0	M0	77.61	Alive
78	F	70	no	ccRCC	I	II	6×5×4	T1b	N0	M0	77.51	Alive
79	M	65	no	ccRCC	III	II	8×7×6	T3a	N0	M0	10.2	Dead

Table S4. siRNA sequence used in the study	
siRNA	Sequence
METTL14-1	S: GCAAAGATGAGCAGAGAGAAATTGCT
	AS: AGCAATTTCTCTCTGCTCATCTTTG
METTL14-2	S: GGGAGCTCATCAGGCTAAAGGATGA
	AS: TCATCCTTTAGCCTGATGAGCTCCC
ESRP2	S: AGCCCGAGGTGATAAAGCA
	AS: TGCTTTATCACCTCGGGCT
YTHDC1	S: CGACCAGAAGATTATGATA
	AS: GCTGGTCTTCTAATACTAT

Table S5. Primers used in RT-qPCR	
Primers	Sequence
METTL3	F: ATCCCCAAGGCTTCAACCAG
	R: GCGAGTGCCAGGAGATAGTC
METTL14	F: TCCCCATAATGATTACTGC
	R: CTGATGTCAAAGGCTTCTAT
WTAP	F: CTTCCCAAGAAGGTTCGATTGA
	R: TCAGACTCTCTTAGGCCAGTTAC
ALKBH5	F: GGCCGTATGCAGTGAGTGATT
	R: TGTCCGTGTCCTTCTTTAGCG
FTO	F: TCAGCAGTGGCAGCTGAAAT
	R: CTTGGATCCTCACCACGTCC
Lnc-LSG1	F: CTTGTGCCTCAGAATCATCATAGAC
	R: CCAGAAGGTGTTAATCATCATCACT
ESRP2	F: TGGTGTGGCCCTCTGTCTCAAC
	R: GCCCCCTGCAATCTTTACGAA
YTHDC1	F: CACCAGAGACCAGGGTATTTAAAGGATC
	R:CATTCCTTGCCAAGGTGGTGGTGGTCCCAT
GAPDH	F: GTGAAGCAGGCGTCGGA
	R: AGCCCCAGCGTCAAAGG

Table S6. The m6A level of lncRNAs downregulated in 786-O and Caki-1 shMETTL14-1 cells

lnc-SKIL-3	lnc-PHLDA1-1
OTUD6B-AS1	lnc-ANKRD55-6
lnc-IL17B-2	NCK1-DT
lnc-TMEM160-1	lnc-CARHSP1-1
lnc-ZFC3H1-10	lnc-SMARCC1-3
lnc-CA8-13	PCBP1-AS1
lnc-KLF10-2	lnc-TCFL5-6
lnc-UBE2Q1-2	lnc-FAM136A-1
lnc-FAM84B-20	lnc-SPOCD1-2
FGD5-AS1	lnc-EIF2AK4-6
lnc-EBF1-4	lnc-LBR-4
lnc-LGALS3BP-2	lnc-RFNG-2
lnc-PDE4D-7	lnc-SLC25A30-4
TUG1	lnc-TRIM37-1
lnc-PCDH15-2	lnc-JUNB-1
DDIT4-AS1	lnc-C1orf141-2
lnc-ZNF121-1	lnc-SLC25A32-4
lnc-GTF2A2-6	lnc-PFDN2-1
lnc-FAM92B-6	lnc-FSCN2-3
lnc-FOXS1-2	lnc-STAT1-2
VCAN-AS1	lnc-MRPL24-2
lnc-PRELID3B-3	lnc-SLC45A3-2
lnc-TAF12-5	lnc-PPP5D1-1
lnc-LSG1-4	NORAD
lnc-FAM166A-3	lnc-BAZ2A-2
lnc-MFSD8-8	lnc-RAB3IL1-2
NEAT1	lnc-CLMP-3
lnc-STPG4-3	lnc-RPL17-2
lnc-SLC43A3-1	lnc-NFS1-1
lnc-EMC1-1	lnc-ZBED5-4
lnc-MGLL-5	lnc-CA1-3
lnc-TMEM189-UBE2V1-4	lnc-TLDC1-2
lnc-CEP152-1	lnc-ARHGEF2-3
lnc-ZDHHC7-2	lnc-MAP4K2-3
lnc-CDC42BPB-4	MALAT1
lnc-RNF39-9	lnc-LTBP3-2
lnc-CCDC57-6	lnc-ISG15-5
lnc-ARID2-11	lnc-PADI2-1
lnc-EEF1E1-2	lnc-TSC1-1
lnc-CTSZ-7	lnc-S100A11-2
lnc-SHANK2-5	lnc-SAMD11-14
lnc-TXNDC16-3	lnc-PASK-2
PSMA3-AS1	lnc-C8orf89-3
lnc-ANKUB1-2	lnc-C9orf64-1
lnc-TFEC-5	lnc-MOS-1
lnc-TRPA1-2	lnc-PDGFB-4
lnc-ITGA11-6	lnc-SKAP2-6
lnc-DCAF1-1	lnc-ITGB1BP2-2
lnc-FRMD5-1	
lnc-SUMF1-18	
lnc-HNRNPU-1	
lnc-PUM2-3	
lnc-C5orf60-3	
lnc-TNPO2-1	
lnc-ENPP1-5	
LINC00958	
lnc-CALCRL-2	
lnc-SAMD11-13	
lnc-PAQR7-1	

Abbreviations list	
**Abbreviations**	**Full name**
ccRCC	Clear cell renal carcinoma
m6A	N6-methyladenosine
METTL14	methyltransferase 14
METTL3	methyltransferase 3
WTAP	WT1 associated protein
FTO	fat mass and obesity associated
ALKBH5	alkB homolog 5
TCGA	The Cancer Genome Atlas
CNVs	copy number variations
OS	overall survival
DFS	disease-free survival
lncRNA	long non-coding RNA
MeRIP-seq	methylated RNA immunoprecipitation sequencing
ESRP2	epithelial splicing regulatory protein 2
YTHDC1	YTH domain containing 1
IHC	immunohistochemistry
FISH	fluorescence in situ hybridization
HE	hematoxylin and eosin staining
CHX	cycloheximide
CQ	chloroquine
FGFR2	fibroblast growth factor receptor 2
