# Supplementary material for: METTL14-mediated Lnc-LSG1 m6A modification inhibits clear cell renal cell carcinoma metastasis via regulating ESRP2 ubiquitination
Source: Mol Ther Nucleic Acids. 2021 Dec 17;27:547–61. doi: 10.1016/j.omtn.2021.12.024 (PMC8738955; doi:10.1016/j.omtn.2021.12.024)
Supplement: Document S2. Article plus supplemental information [file mmc2.pdf]

Molecular Therapy
Nucleic Acids
Original Article

METTL14-mediated Lnc-LSG1 m6A modification inhibits clear cell renal cell carcinoma metastasis via regulating ESRP2 ubiquitination

Danyang Shen,[1,2,4,5] Lifeng Ding,[1,4,5] Zeyi Lu,[1] Ruyue Wang,[1] Chenhao Yu,[1] Huan Wang,[1] Qiming Zheng,[1] Xuliang Wang,[1] Wanjiang Xu,[1] Haifeng Yu,[1] Liwei Xu,[1] Mingchao Wang,[1] Shicheng Yu,[1] Shibin Zhu,[1] Jun Qian,[3] Liqun Xia,[1] and Gonghui Li[1]

[1]Department of Urology, Sir Run Run Shaw Hospital, Zhejiang University School of Medicine, Hangzhou 310016, China; [2]Department of General Surgery, The First Affiliated Hospital of Soochow University, Suzhou 215006, China; [3]State Key Laboratory of Modern Optical Instrumentations, Centre for Optical and Electromagnetic Research, College of Optical Science and Engineering, Zhejiang University, Hangzhou 310058, China

Clear cell renal cell carcinoma (ccRCC) is the most lethal urological cancer and is characterized by a high rate of metastasis and relapse. N6-Methyladenosine (m^{6}A) is implicated in various stages of cancer development. However, a thorough understanding of m^{6}A-modified lncRNAs in ccRCC is lacking. The results showed that METTL14 had decreased expression in ccRCC tissues. In addition, the expression of METTL14 was negatively correlated to the prognosis, stage, and ccRCC tumor grade. The silencing of METTL14 was shown to significantly increase metastasis *in vitro* and *in vivo*. High-throughput methylated RNA immunoprecipitation sequencing (MeRIP-seq) showed that the m^{6}A levels of Lnc-LSG1 could be regulated by METTL14. Lnc-LSG1 can directly bind to ESRP2 protein and promote ESRP2 degradation via facilitating ESRP2 ubiquitination. However, m^{6}A modification on Lnc-LSG1 can block the interaction between Lnc-LSG1 and ESRP2 via the m^{6}A reader, YTHDC1. Taken together, our findings unraveled the novel mechanism of METTL14 inhibiting ccRCC progression, and explored the correlation between m^{6}A and lncRNA in ccRCC for the first time.

INTRODUCTION

Renal cell carcinoma (RCC) accounts for 4% of all malignancies and is the most lethal urological cancer in the United States.[1] Approximately 90% of RCCs are clear cell RCC (ccRCC) which is characterized by a high rate of metastasis and relapse. Nephrectomy is considered as the gold standard for RCC treatment. However, about 30% of ccRCC patients present with metastasis at the initial diagnosis. In addition, up to one-third of patients with early-stage ccRCC develop metastasis following nephrectomy, thus poor prognosis.[2] Therefore, exploring the mechanisms underlying metastasis and identifying new biomarkers for predicting ccRCC progression may give insights into the development of highly efficacious therapies.

N6-Methyladenosine (m^{6}A) is the most prevalent mRNA modification in eukaryotic cells.[3] It is a dynamic and reversible process involved in distinct mRNA metabolism phases, including splicing,[4] translation,[5] nuclear export,[6] and stability.[7] It is catalyzed by an m^{6}A methyltransferase complex composed of methyltransferase 3 (METTL3), methyltransferase 14 (METTL14), and WT1-associated protein (WTAP). The m^{6}A methylation can be reversed by m^{6}A demethylases, including fat mass and obesity-associated protein (FTO) and alkB homolog 5 (ALKBH5). The methylation is detected by m^{6}A “reader” proteins, which include YTH domain proteins, including YTH m^{6}A RNA binding protein 1–3 (YTHDF1-3) and YTH domain containing 1–2 (YTHDC1-2), heterogeneous nuclear ribonucleoproteins, and insulin-like growth factor-2 mRNA-binding proteins.[8] Recently, numerous studies have implicated the dysregulation of m^{6}A modification in several pathological processes.[9,10] A previous study evaluated the prognostic value of m^{6}A readers and writers in ccRCC by analyzing RNA sequencing and copy number variations (CNVs) data from The Cancer Genome Atlas (TCGA) database obtained from 528 ccRCC patients. The results revealed that CNVs in the m^{6}A readers and writers were associated with poorer overall survival (OS) and disease-free survival (DFS).[11] However, the detailed molecular mechanisms through which m^{6}A modification regulates ccRCC progression have not been elucidated.

Previous studies mainly focused on how the m^{6}A modifications determined the fate of mRNAs. Recent accumulating evidence has shed

Received 22 June 2021; accepted 15 December 2021;
https://doi.org/10.1016/j.omtn.2021.12.024.

[4]These authors contributed equally

[5]Senior authors

Correspondence: Gonghui Li, PhD, Department of Urology, Sir Run Run Shaw Hospital, Zhejiang University School of Medicine, 3 Qingchun Road, Hangzhou, China, 310016.
E-mail: 3193119@zju.edu.cn
Correspondence: Liqun Xia, PhD, Department of Urology, Sir Run Run Shaw Hospital, Zhejiang University School of Medicine, 3 Qingchun Road, Hangzhou, China, 310016.
E-mail: xialiqun@zju.edu.cn

This is an open access article under the CC BY license (http://creativecommons.org/licenses/by/4.0/).

light on the correlation between m^{6}A methylation and noncoding RNAs (ncRNAs).[12,13] An m^{6}A mapping study reported that m^{6}A methylation was also extensively present in long noncoding RNAs (lncRNAs).[14] Furthermore, m^{6}A was shown to influence cancer progression by regulating the metabolism and functions of lncRNAs.[7,15,16] This suggests that m^{6}A-modified lncRNAs may play a crucial role in cancer. To date, no study has explored the role of m^{6}A modification and lncRNAs in ccRCC.

In this study, we found that METTL14 functions as a tumor suppressor in ccRCC metastasis using patients' samples from our own cohort and a ccRCC tissue microarray, as well as *in vitro* and *in vivo* experiments. By performing high-throughput methylated RNA immunoprecipitation sequencing (MeRIP-seq) and Transwell assay, we identified Lnc-LSG1 as the downstream target of anti-metastatic function of METTL14. Further experiments demonstrated that m^{6}A on Lnc-LSG1 blocked the interaction between Lnc-LSG1 and epithelial splicing regulatory protein 2 (ESRP2) protein via YTHDC1, thus protecting ESRP2 from ubiquitinated degradation. Our findings expand the understanding of the role and underlying mechanisms of METTL14-mediated lncRNA m^{6}A modification in ccRCC progression and prognosis, and provide a new insight into ccRCC metastasis from the aspect of the epigenetic modification of noncoding RNAs.

RESULTS

Decreased METTL14 expression was associated with poor prognosis

The total m^{6}A levels in nine pairs of ccRCC tumor tissues and the paired peritumor tissues were determined using the m^{6}A RNA methylation quantification kit to investigate the potential role of m^{6}A modification in ccRCC. The results revealed that the m^{6}A levels were significantly decreased in the ccRCC tumor tissues (Figure 1A). The decreased m^{6}A levels in the ccRCC tissues were probably due to a dysregulation of the writers and erasers since the m^{6}A modification is mainly catalyzed by m^{6}A writers and erasers.

The expression profiles of key m^{6}A writers (METTL3, METTL14, and WTAP) and erasers (ALKBH5 and FTO) in 50 pairs of ccRCC tissues obtained from patients who had undergone nephrectomy at Sir Run Run Shaw Hospital (SRRSH) was determined. The qRT-PCR assay showed that METTL14, an essential component of the m^{6}A methyltransferase complex,[17] was significantly downregulated in ccRCC. However, there were no significant differences in the expression of METTL3, WTAP, ALKBH5, and FTO (Figure 1B). Moreover, protein levels of METTL14 were significantly decreased in the ccRCC tissues than in their adjacent normal tissues (Figure 1C). Subsequently, results showing decreased METTL14 protein expression were validated in 40 ccRCC samples obtained from SRRSH cohort and in a tissue microarray constructed from 79 ccRCC samples based on immunohistochemistry (IHC) staining (Figures 1D and 1E). The results revealed that METTL14 was significantly decreased in the ccRCC tissues.

Furthermore, patients with low expression of METTL14 showed poorer OS, suggesting that METTL14 plays a tumor-suppressor role in ccRCC (Figure 1F). These results were consistent with the ccRCC data mining results from Database: UALCAN (http://ualcan.path.uab.edu/), which showed that METTL14 had decreased expression in ccRCC tissues (Figure S1A). Moreover, levels of METTL14 were negatively correlated to the tumor stage, grade, and nodal metastasis status (Figures S1B and S1C). Finally, the Kaplan-Meier survival analysis of data obtained from Database: GEPIA (http://gepia.cancer-pku.cn/) also showed that decreased expression of METTL14 was associated with poor OS and DFS (Figures S1D and S1E). Taken together, these data suggest that METTL14 might be involved in ccRCC progression and is a potential prognostic indicator in ccRCC patients.

METTL14 inhibited metastasis in ccRCC via m^{6}A modification

We generated METTL14-overexpressing and knockdown cell models in 786-O, Caki-1, and OSRC-2 cells to evaluate whether METTL14 was negatively correlated to ccRCC progression. The overexpression or knockdown of METTL14 was confirmed by determining the mRNA and protein levels (Figures 2A, 2B, and S2A). The proliferative and metastatic abilities were assessed by using CCK8 and Transwell assays, respectively. Results of the CCK8 assay showed that METTL14 did not affect cell proliferation (Figure S2B). However, METTL14 knockdown significantly increased the migration and invasion ability of ccRCC cells. In contrast, overexpression of METTL14 significantly decreased the migration and invasion ability of the ccRCC cells (Figures 2C and S2C). These results suggest that METTL14 is a negative regulator in ccRCC metastasis. Furthermore, the wound healing assay showed similar results (Figures 2D and S2D).

Moreover, we constructed plasmids expressing wild-type METTL14 (METTL14-WT) and mutant METTL14 (METTL14-R298P; R298 is critical for the target recognition of the methyltransferase complex[17,18]) to determine whether the effect of METTL14 on metastasis was dependent on its ability to recognize m^{6}A targets. As shown in Figure 2E, the ectopic expression of METTL14-WT, but not METTL14-R298P, reversed the increased migration and invasion induced by METTL14 silencing. These observations suggested that METTL14 inhibited *in vitro* migration and invasion in ccRCC in an m^{6}A-dependent manner.

Furthermore, we established stable METTL14 knockdown in luciferase-expressing OSRC-2 cells to investigate the anti-metastatic effect of METTL14 in ccRCC *in vivo*. The control and METTL14 knockdown cells were orthotopically injected into the renal capsule of BALB/c nude mice. The metastatic ability was then determined using a bioluminescence imaging system after 6 weeks. The METTL14 knockdown group showed increased cancer metastasis (Figure 2F) and a higher metastatic ratio than the control group (Figure 2G). Subsequently, we established a ccRCC lung metastasis model by injecting METTL14 knockdown and corresponding control cells into the tail vein of BALB/c nude mice. The silencing of METTL14 was shown to significantly promote lung metastases, as shown by increased bioluminescent signals in the lungs (Figure 2H). Moreover, hematoxylin and eosin (H&E) staining showed a higher

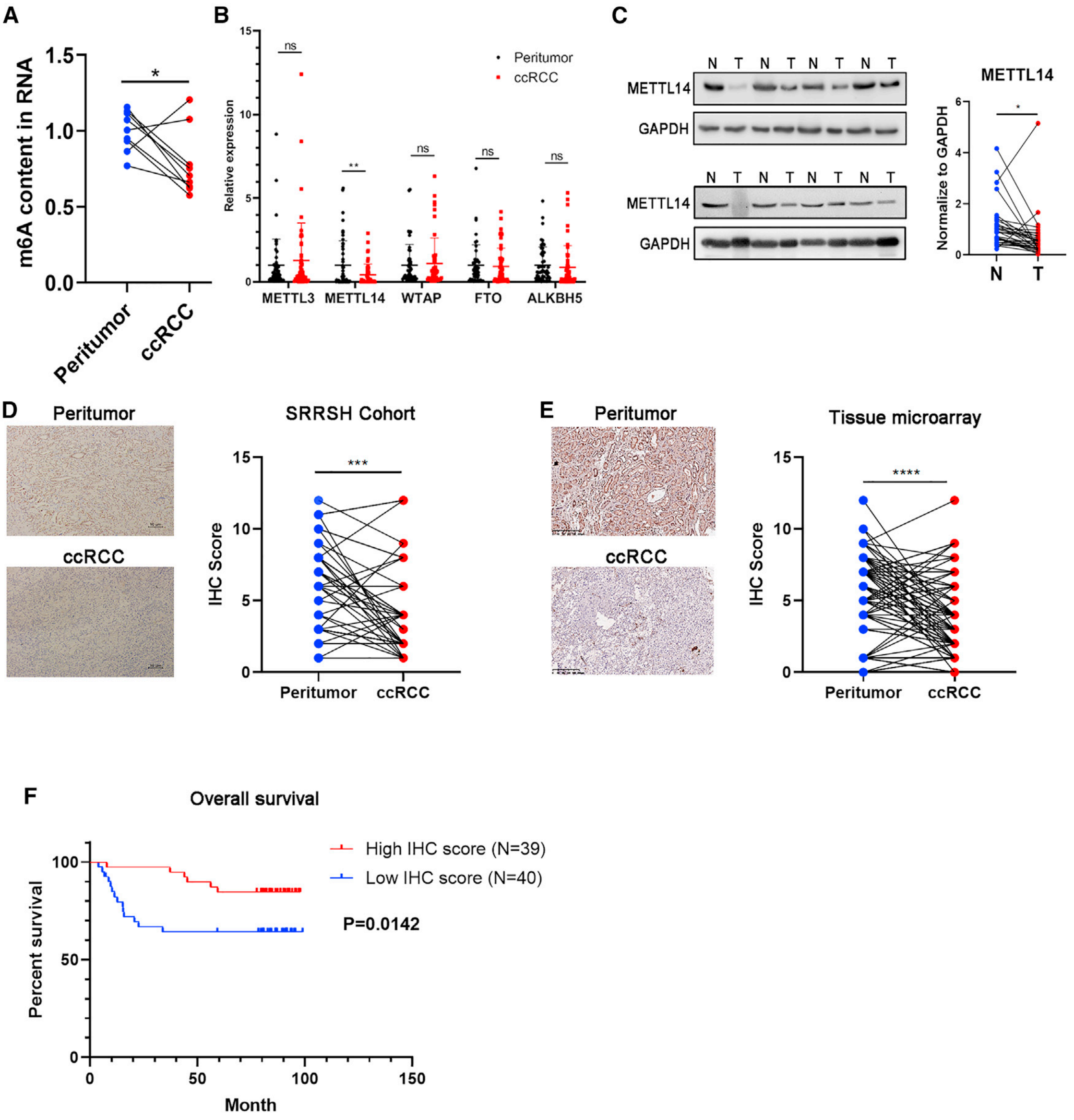

Figure 1. Decreased METTL14 expression correlates to poor prognosis in ccRCC patients.

(A) The m6A levels of the total extracted RNA from nine pairs of ccRCC tissues. (B) The qRT-PCR assay results showing mRNA levels of METTL3, METTL14, WTAP, FTO, and ALKBH5 compared with GAPDH controls in 50 pairs of ccRCC tissues. (C) The protein levels of METTL14 in 32 pairs of ccRCC tissues. N, normal tissue; T, tumor tissue. (D) Representative IHC images of METTL14 in 40 ccRCC tissues from the SRRSH cohort. The IHC scores of each sample were calculated. Dots, IHC score; lines, pairs of normal and tumor tissues. Scale bars represent 10 μm. SRRSH, Sir Run Run Shaw Hospital. (E) Representative IHC images of METTL14 in tissue microarrays obtained from 79 pairs of ccRCC tissues and their corresponding IHC scores. Dots, IHC score; lines, pairs of normal and tumor tissues. Scale bars represent 200 μm. (F) Kaplan-Meier survival curves showing overall survival of 79 ccRCC patients based on the IHC score. *$p < 0.05$, **$p < 0.01$, ***$p < 0.001$, ****$p < 0.0001$; ns, not significant. Data are representative of three independent experiments.

number of metastatic tumor nodules in the lungs of the METTL14 knockdown group than in the control group (Figure 2I). We also detected the effect of METTL14 on tumor proliferation. The results showed that the volume of the tumors was nearly the same in the shMETTL14-1 and control groups (Figure S2E), consistent with the CCK8 results.

Lnc-LSG1 was identified as a downstream target of METTL14

METTL14 is a component of the m^{6}A methyltransferase. Knockdown of METTL14 leads to decreased m^{6}A levels. To date, no studies have investigated m^{6}A methylation of lncRNAs in ccRCC. This study investigated whether lncRNAs m^{6}A methylation mediated the anti-metastatic effect of METTL14. Therefore, we

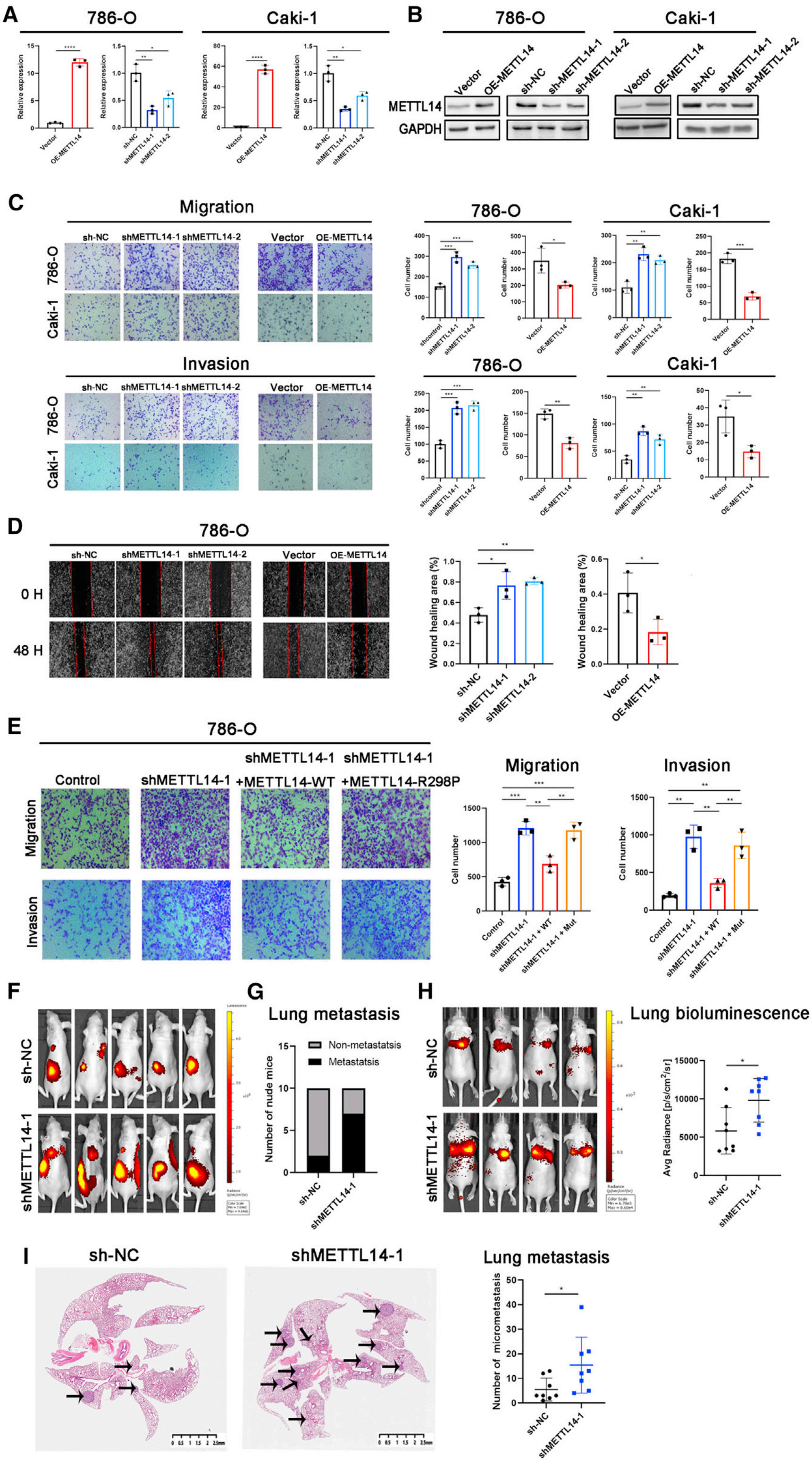

Figure 2. METTL14 inhibited ccRCC metastasis via m^{6}A modification.

(A and B) qRT-PCR and western blotting analysis were used to confirm overexpression and knockdown of METTL14 in 786-O and Caki-1 cells. (C and D) Transwell assay and wound healing assay were performed to detect the migratory and invasive abilities of ccRCC cells after METTL14 knockdown or overexpression. Magnification, 100×. (E) Transwell assay showed that METTL14-WT, but not METTL14-R298P, could reverse the effect of METTL14 knockdown on 786-O cell migration and invasion. Magnification, 100×. WT, wild type; Mut, R298P mutant. (F) OSRC-2 shMETTL14-1 or sh-NC cells labeled with luciferase expression were injected into the renal capsule of the mice (n = 10 per group). Representative bioluminescent images showing systemic metastasis. (G) The ratio of lung metastasis was higher in the shMETTL14-1 group (7/10) than in the sh-NC group (2/10). (H) Luciferase-tagged OSRC-2 shMETTL14-1 or sh-NC cells were injected into mice tail vein (n = 8 per group). Representative bioluminescent images showing lung metastasis. Quantification of the bioluminescent signal intensities (photons/s/cm^2/sr) in the lungs was carried out after 6 weeks. (I) Micrometastasis in lungs harvested from the shMETTL14-1 and sh-NC groups were evaluated by H&E staining. Scale bars represent 2.5 mm. Representative H&E staining images of the lung sections are shown. *$p < 0.05$, **$p < 0.01$, ***$p < 0.001$; ns, not significant. Data are presented as the mean ± SD of at least three independent experiments.

performed MeRIP-seq in control and stable METTL14-knockdown 786-O and Caki-1 cells. Levels of m^{6}A in 107 lncRNAs were decreased after METTL14 knockdown in both 786-O and Caki-1 cells, including MALAT1, XIST, and NEAT1. The downregulation of MALAT1, XIST, and NEAT1 has been reported in other studies[7,15,16] (Figure 3A; Table S6). Among these, we selected Lnc-IL17B-2, Lnc-ZNF121-1, Lnc-LSG1-4:2, and Lnc-ENPP1-5 for further investigation (logFC < −1, p < 0.05) (Figure 3A). Results of the m^{6}A levels of the four lncRNAs after METTL14 knockdown were validated using MeRIP and qRT-PCR assay. As shown in Figure 3B, only Lnc-ZNF121-1 and Lnc-LSG1-4:2 exhibited decreased m^{6}A levels after METTL14 knockdown in the 786-O and Caki-1 cells, with Lnc-LSG1-4:2 (Lnc-LSG1) exhibiting the highest decrease in m^{6}A levels. The Integrative Genomics Viewer plots also showed a decreased m^{6}A peak at the Lnc-LSG1 transcripts (Figure 3C). The 5′ and 3′ rapid amplification of cDNA ends analysis showed that Lnc-LSG1 had a full length of 980 nt (Figure S2F). Furthermore, we compared the expression of Lnc-LSG1 with several highly expressed lncRNAs in ccRCC to investigate the abundance of Lnc-LSG1.[19–21] The qRT-PCR assay revealed that the expression of Lnc-LSG1 was higher than MALAT1 in the 786-O cells, and UCA1 in the 786-O and OSRC-2 cells. These results suggested a high abundance of Lnc-LSG1 in the ccRCC cells (Figure S2G). Bioinformatics analysis using the online database Coding Potential Calculator 2.0 (CPC 2.0, http://cpc2.gao-lab.org/index.php) showed that Lnc-LSG1 could not code for proteins[22] (Figure S2H). The Transwell assay showed that knockdown of Lnc-LSG1 decreased the migration and invasion ability of ccRCC cells. In contrast, overexpression of Lnc-LSG1 increased the migration and invasion ability of ccRCC cells, suggesting that Lnc-LSG1 could be a metastasis-related lncRNA (Figures 3D, S2I, and S2J). Moreover, knockdown of Lnc-LSG1 effectively reversed the increased metastatic ability of METTL14 knockdown cells (Figure 3E). We also investigated the effect of Lnc-LSG1 on cell proliferation using *in vitro* and *in vivo* assays. The results showed that Lnc-LSG1 did not influence the proliferation of ccRCC cells (Figures S2K and S2L). Therefore, Lnc-LSG1 was considered as a candidate substrate of METTL14 for further investigation.

Lnc-LSG1 regulates ccRCC metastasis via binding to ESRP2 protein and repressing ESRP2 stability

We explored the functional mechanisms by which Lnc-LSG1 increased ccRCC metastasis. The qRT-PCR and fluorescence *in situ* hybridization (FISH) analysis of the fractionated cytoplasmic and nuclear RNA obtained from 786-O cells showed that Lnc-LSG1 was expressed in the nucleus and the cytoplasm, and mainly prevalent in the cytoplasm (Figures 4A and 4B). LncRNA can activate the transcription of nearby genes in *cis* by promoting chromatin looping from transcriptional enhancers.[23,24] Therefore, we investigated the effect of Lnc-LSG1 on TMEM44, the neighboring protein-coding gene (Figure S3A). There was no significant difference in the expression level of TMEM44 following Lnc-LSG1 knockdown or overexpression (Figure S3B). This suggests that the biological functions of Lnc-LSG1 were not related to the *cis*-regulatory function.

LncRNAs are associated with a plethora of cellular functions, most of which require interaction with one or more proteins. Currently, available information points to an intricate network of protein-lncRNA interactions, whose dysregulation is associated with pathological states.[25] The online Database: catRAPID (http://s.tartaglialab.com/page/catrapid_group)[26] predicted potential proteins that may interact with Lnc-LSG1. Epithelial-specific splicing regulator (ESRP2)[27] was identified as having the highest probability of interacting with Lnc-LSG1 (Figure 4C). The Lnc-LSG1 pull-down and RIP assays with an anti-ESRP2 antibody were used to verify direct binding of Lnc-LSG1 to ESRP2 (Figures 4D and 4E). Furthermore, we constructed a series of truncated Lnc-LSG1 and ESRP2 to determine the exact regions of interaction between Lnc-LSG1 and ESRP2. The Lnc-LSG1 nucleotides (0–300) were shown to be essential in the binding of ESRP2. Moreover, the RNA Recognition Motif 3 of ESRP2 was required for binding Lnc-LSG1 (Figures 4F and 4G). These results confirmed that Lnc-LSG1 is a binding partner of ESRP2.

A previous study reported that ESRP2 knockdown enhanced cell migration in ccRCC.[28] Therefore, we hypothesized that Lnc-LSG1 promoted ccRCC metastasis via ESRP2. Results of the western blotting analysis demonstrated that overexpression of Lnc-LSG1 significantly decreased protein levels of ESRP2. In contrast, Lnc-LSG1 knockdown increased ESRP2 protein levels (Figures 4H and S3C). The qRT-PCR analysis showed that mRNA levels of ESRP2 were not affected by Lnc-LSG1 (Figure S3D). Furthermore, we hypothesized that Lnc-LSG1 promoted degradation of ESRP2 protein based on the fact that lncRNAs modulate protein stability via direct interaction.[24,29] Therefore, we treated ccRCC cells with a protein translation inhibitor, cycloheximide (CHX), to block ESRP2 synthesis. After that, we detected the ESRP2 protein levels using western blotting. The results showed that overexpression of Lnc-LSG1 shortened the half-life of the ESRP2 protein (Figure 4I). Furthermore, the ESRP2 protein levels were significantly increased after treatment with MG132 (a proteasome inhibitor) compared with chloroquine treatment (an autophagy inhibitor) (Figure 4J). These results imply that Lnc-LSG1-induced ESRP2 degradation may be dependent on the ubiquitin-proteasome pathway. Accordingly, overexpression of Lnc-LSG1 significantly increased the ESRP2 ubiquitination levels (Figure 4K). The transwell assay confirmed that ESRP2 knockdown promoted ccRCC migration and invasion (Figures 4L, S3E, and S3F). In addition, impaired metastatic ability in Lnc-LSG1-silenced cells could be restored by knocking down ESRP2 (Figures 4M and S3G), suggesting that ESRP2 was a potential downstream target of Lnc-LSG1. Taken together, these results reveal that Lnc-LSG1 promotes ccRCC metastasis by directly binding to the ESRP2 protein, thus inhibiting its expression through the ubiquitin-proteasome pathway.

METTL14 increases ESRP2 protein stability via Lnc-LSG1

Furthermore, we sought to investigate whether ESRP2 was a downstream target of METTL14 in inhibiting ccRCC metastasis. The anti-m^{6}A RIP assay found that ESRP2 mRNA had no m^{6}A modification. In addition, METTL14 did not regulate ESRP2 m^{6}A levels. Based on these findings, we ruled out the possibility of METTL14 directly

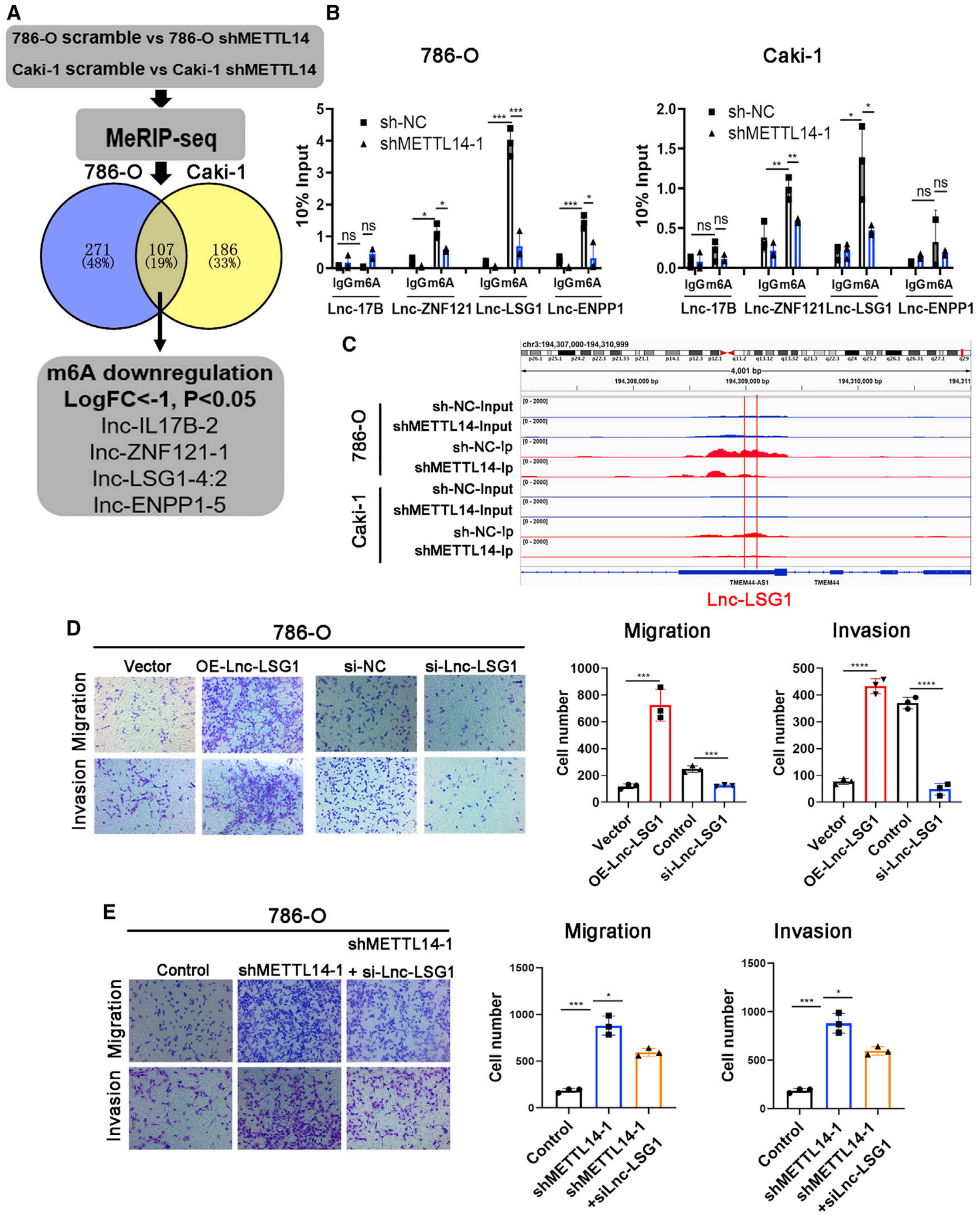

Figure 3. Lnc-LSG1 was identified as a downstream target of METTL14

(A) MeRIP-seq identified four lncRNAs whose m^{6}A levels were significantly decreased following METTL14 knockdown in 786-O and Caki-1 cells. (B) The RIP and qRT-PCR assays showed that m^{6}A could modify Lnc-ZNF121 and Lnc-LSG1 m^{6}A levels. In addition, knockdown of METTL14 could decrease their m^{6}A levels. (C) Integrative Genomics Viewer plots showing a decreased m^{6}A peak at the Lnc-LSG1 transcripts. (D) Transwell assay showed that Lnc-LSG1 could regulate the migration and invasion of the 786-O cells. (E) Inhibition of Lnc-LSG1 suppressed the migratory and invasive abilities of 786-O cells increased by shMETTL14-1. *p < 0.05, **p < 0.01, ***p < 0.001, ****p < 0.0001; ns, not significant. Data are presented as the mean ± SD of at least three independent experiments.

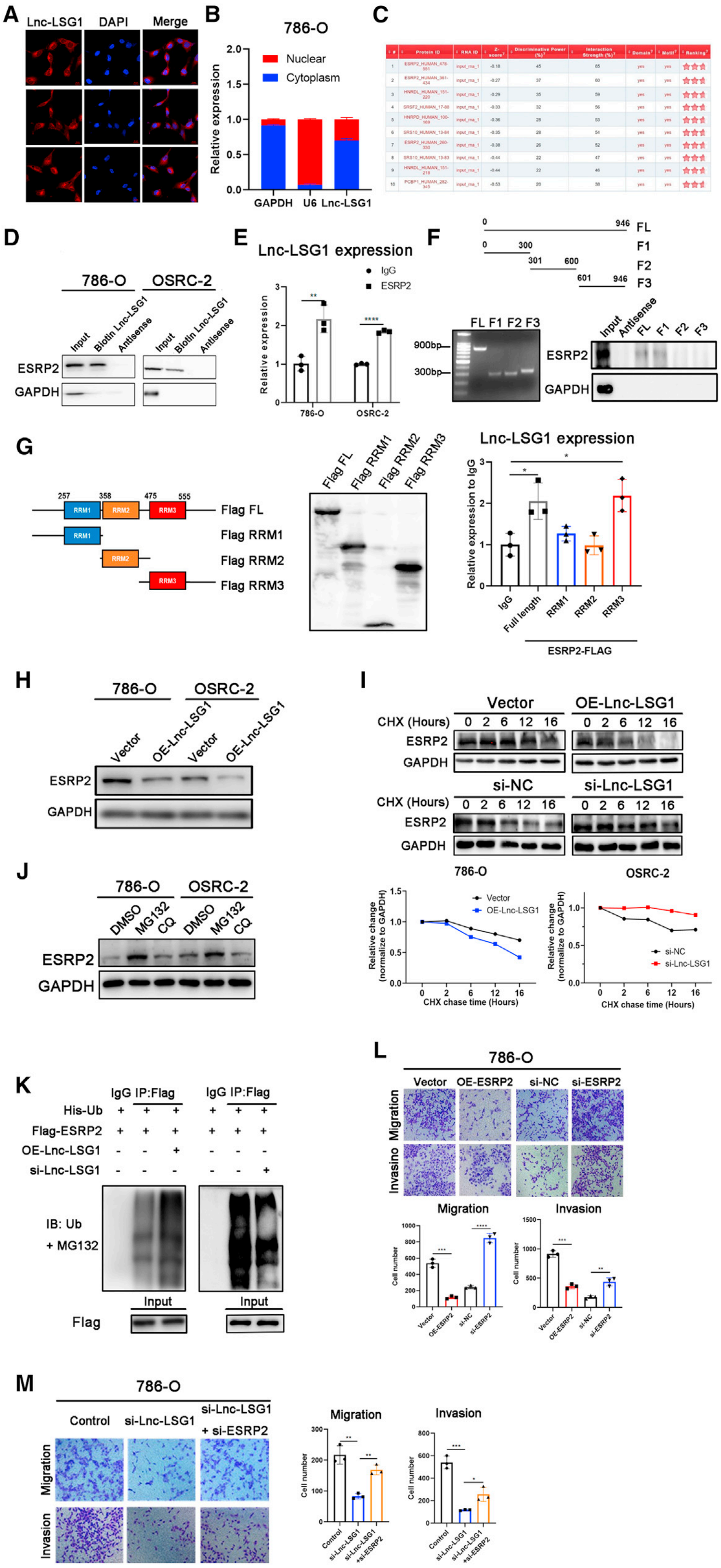

Figure 4. Lnc-LSG1 regulates ccRCC metastasis via binding to ESRP2 protein and repressing ESRP2 stability

(A and B) FISH and qRT-PCR assays showing the intracellular distribution of Lnc-LSG1 in 786-O cells. (C) The catRAPID algorithm was used to predict proteins that could potentially bind to Lnc-LSG1. Data were obtained on 16 March, 2021. (D) RNA pull-down assay followed by western blotting analysis showed that ESRP2 could be precipitated by the Lnc-LSG1 probe but not the antisense probe. (E) RIP assay for the enrichment of Lnc-LSG1 in 786-O and OSRC-2 incubated with IgG or ESRP2 antibody. Lnc-LSG1 was highly enriched in the ESRP2 group compared with the IgG group. (F) RNA pull-down assay with biotin-labeled full-length or truncated Lnc-LSG1, followed by western blotting analysis using ESRP2 and GAPDH antibody. FL, full length. F1, F2, and F3, fragments 1, 2, and 3. (G) RIP assay for flag-tagged full-length or truncated ESRP2 protein, followed by qRT-PCR assay for Lnc-LSG1. (H) Western blotting assay was used to evaluate the effect of Lnc-LSG1 on ESRP2 protein expression. RRM, RNA recognition motif. (I) The western blotting assay showed that cells treated with CHX (50 μg/mL) for the indicated hours shortened the ESRP2 protein half-life in 786-O cells overexpressing Lnc-LSG1. On the other hand, the ESRP2 protein half-life was increased in OSRC-2 cells with Lnc-LSG1 silencing. CHX, cycloheximide. (J) Western blotting assay showing expression of the ESRP2 protein after treatment with MG132 (50 μg/mL for 6 h) or CQ (chloroquine) (20 μM for 24 h). (K) Cells were treated with MG132 (50 μg/mL) for 6 h after transfection. Flag-IP followed by western blotting assay was performed to detect the ubiquitination levels of ESRP2 protein after Lnc-LSG1 overexpression or knockdown in 786-O cells. Ub, ubiquitin; IB, immunoblotting. (L) Transwell assay showed that ESRP2 could regulate the migration and invasion of 786-O cells. (M) Inhibition of ESRP2 suppressed the inhibitory effect of Lnc-LSG1 knockdown on the migratory and invasive ability of the 786-O cells. $^{*}p < 0.05$, $^{**}p < 0.01$, $^{***}p < 0.001$, $^{****}p < 0.0001$. The error bars represent ± SD of three biological replicates.

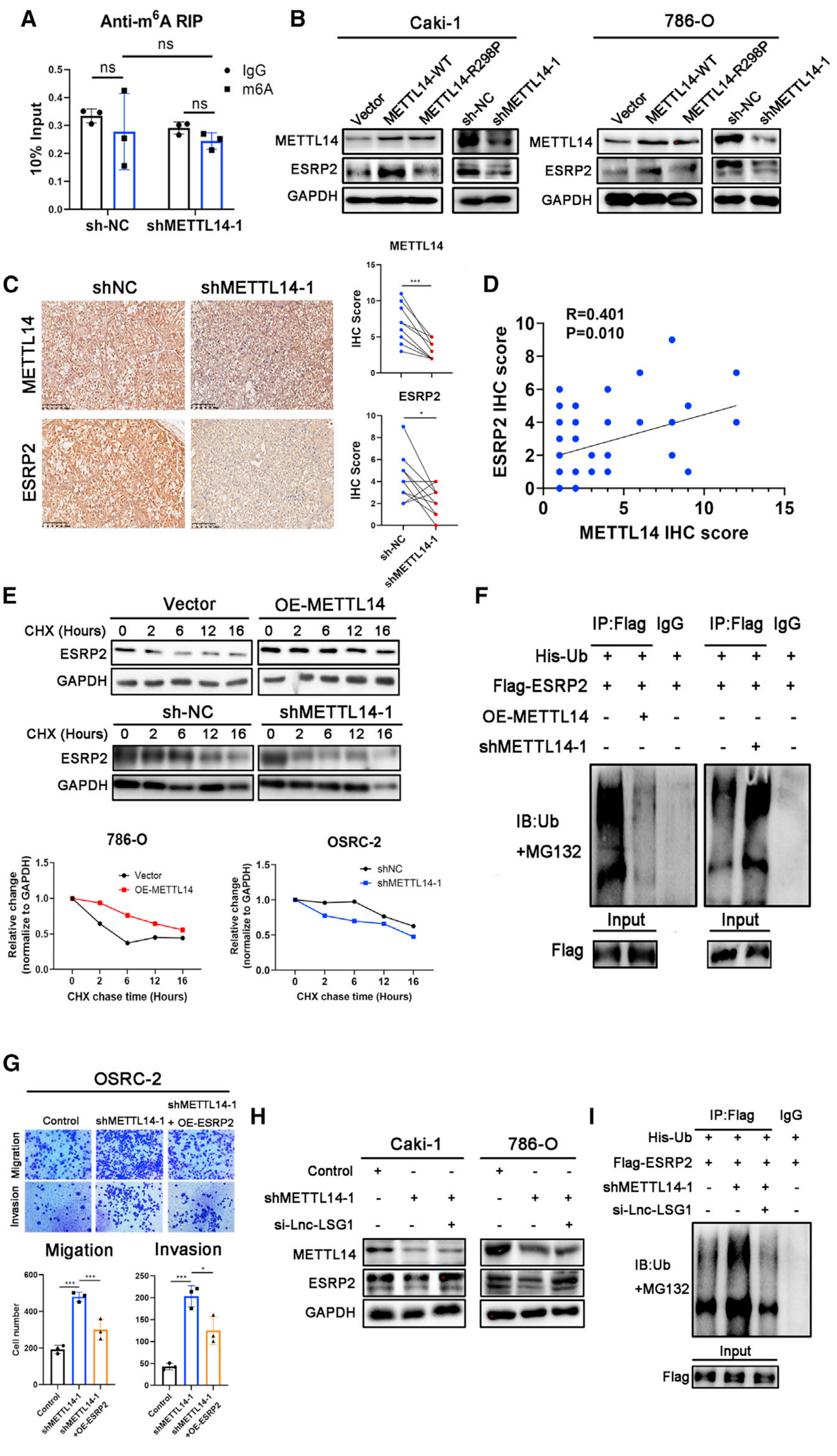

Figure 5. METTL14 increases ESRP2 protein stability via Lnc-LSG1

(A) Anti-m^{6}A RIP assay showed that ESRP2 mRNA has no m^{6}A modification, and METTL14 cannot regulate m^{6}A level of ESRP2 mRNA. (B) Western blotting assay was performed to analyze the effect of METTL14 on the ESRP2 protein. (C) Immunohistochemical staining results of the orthotopic tumor sections stained with METTL14 and ESRP2 antibodies. Scale bars represent 200 μm. (D) In the IHC analysis of 40 ccRCC samples obtained from SRRSH cohort, the scatterplot shows the correlation between the expression of METTL14 and ESRP2 proteins in ccRCC. (E) Western blotting assay showed that the half-life of the ESRP2 protein was prolonged in 786-O OE-METTL14 cells treated with CHX (50 μg/mL) for the indicated hours. In contrast, the half-life was shortened in OSRC-2 shMETTL14-1 cells. (F) Cells were subjected to MG132 after transfection. Flag-IP followed by western blotting assay was conducted to detect the ubiquitination levels of ESRP2 protein in 786-O OE-METTL14 and shMETTL14-1 cells. (G) Overexpression of ESRP2 can abolish the metastatic ability of OSRC2 cells induced by METTL14 knockdown. (H and I) Knockdown of Lnc-LSG1 can abolish the effect of METTL14 knockdown on ESRP2 expression (H) and ubiquitination levels (I). *$p < 0.05$, ***$p < 0.001$. The error bars represent ± SD of three biological replicates.

modifying ESRP2 via m^{6}A (Figure 5A). The qRT-PCR assay showed that METTL14 did not affect the mRNA expression of ESRP2 (Figure S4A). Western blotting analysis indicated that the ectopic expression of METTL14-WT, but not METTL14-R298P, increased ESRP2 protein levels, suggesting that METTL14 promotes ESRP2 protein expression through regulating m^{6}A methylation (Figure 5B). Conversely, ESRP2 protein expression was inhibited in shMETTL14 cell lines (Figure 5B). Moreover, IHC staining showed decreased expression of ESRP2 in orthotopic tumors injected with METTL14 knockdown cells (Figure 5C). We also used 40 ccRCC samples in Figure 1D for IHC analysis and calculated the ESRP2 protein IHC score. Then, Pearson's correlation analysis showed that the METTL14 protein expression was positively correlated with ESRP2 protein expression ($r = 0.401$, $p = 0.010$; Figure 5D).

In addition, METTL14 was shown to extend the half-life of ESRP2 protein (Figure 5E) and reduce its ubiquitination level (Figure 5F). However, shMETTL14 showed opposite effects (Figures 5E and 5F). Importantly, ESRP2 reversed the increased metastatic ability induced by the knockdown of METTL14 in ccRCC cells (Figures 5G and S4B). These findings suggest that METTL14 inhibits ccRCC metastasis by decreasing ESRP2 ubiquitination and increasing ESRP2 protein levels.

Furthermore, we transfected the Lnc-LSG1 smart silencer into shMETTL14 cell lines to determine whether METTL14 regulated ESRP2 protein levels through Lnc-LSG1. Western blotting assay demonstrated that the METTL14 knockdown-induced downregulation of ESRP2 protein could be restored by knockdown of Lnc-LSG1 (Figure 5H). Furthermore, knockdown of Lnc-LSG1 decreased the ubiquitination of ESRP2 induced by shMETTL14 (Figure 5I). In summary, these results indicate that METTL14 increases ESRP2 protein stability via Lnc-LSG1.

METTL14 inhibits the interaction between ESRP2 and Lnc-LSG1 through YTHDC1

Furthermore, we investigated the detailed mechanism of how METTL14 regulated ESRP2 protein degradation through Lnc-LSG1. METTL14 and Lnc-LSG1 show opposing effects on the ESRP2 protein. In addition, METTL14 regulates m^{6}A methylation of Lnc-LSG1. Therefore, we hypothesized that METTL14 could inhibit the function of Lnc-LSG1 in an m^{6}A-dependent manner. The qRT-PCR assay showed that METTL14 did not regulate the expression of Lnc-LSG1 (Figure S4C). Most studies showed that m^{6}A methylation regulates lncRNA function by modulating lncRNA stability.[30,31] However, other authors reported that m^{6}A methylation might also be directly involved in lncRNA-RNA and lncRNA-protein interactions. For example, a recent study by Yoneda et al. reported that m^{6}A modification in lncRNA pcnRNA-D abolished the direct binding of pncRNA-D to the protein TLS through an m^{6}A reader YTHDC1.[32] The Lnc-LSG1 facilitates ESRP2 ubiquitination through direct binding. In addition, METTL14 reduces ESRP2 ubiquitination. Based on these facts, we hypothesized that METTL14 inhibited the binding of ESRP2 and Lnc-LSG1 by promoting YTHDC1 binding to the m^{6}A sites on Lnc-LSG1. This is similar to the effect of m^{6}A-YTHDC1 on the interaction between pcnRNA-D and TLS protein.[32]

The anti-YTHDC1 RIP assay showed that YTHDC1 had significantly increased binding to the Lnc-LSG1 than the IgG control (Figure 6A). Furthermore, anti-YTHDC1 RIP assay showed that the interaction between YTHDC1 and Lnc-LSG1 could be regulated by METTL14 (Figure 6B), and qRT-PCR assay showed that YTHDC1 did not affect mRNA expression of ESRP2 (Figure S4D), suggesting that YTHDC1 was the m^{6}A reader of Lnc-LSG1 m^{6}A methylation. The anti-ESRP2 RIP assay showed increased ESRP2/Lnc-LSG1 interaction in shMETT14 cells and decreased interactions in the OE-METTL14 cells (Figures 6C and 6D). However, METTL14-R298P had no effect on ESRP2/Lnc-LSG1 interaction (Figure 6E), which suggested that the inhibitory function of METTL14 on Lnc-LSG1/ESRP2 interaction is mediated by m^{6}A. Moreover, siYTHDC1 enhanced the binding of Lnc-LSG1 to ESRP2 protein and abrogated the inhibited ESRP2/Lnc-LSG1 interaction induced by METTL14 overexpression (Figure 6D). These results provide evidence that METTL14 regulates the binding between ESRP2 and Lnc-LSG1 via YTHDC1.

M^{6}A modification tends to occur in an RRACH (R: A or G; H: A, C, or U) consensus sequence,[33] especially the GGACU.[34–36] To identify the adenosine residue(s) on Lnc-LSG1 responsible for YTHDC1 binding, we searched for the RRACH motif in nucleotides 0 to 300 of Lnc-LSG1, required for the ESRP2/Lnc-LSG1 interaction (Figure 4F). The results showed four RRACH motifs and only one GGACU sequence in nucleotides 0 to 300 (Figure 6F). We then generated a 25 nt biotinylated RNA probe around GGACU with or without m^{6}A methylation (probe m^{6}A and probe A) to investigate the function of m^{6}A methylation in the GGACU sequence (Figure 6G). Subsequently, we conducted an RNA pull-down assay using the two probes. Furthermore, we determined the precipitated protein levels of YTHDC1 and ESRP2. The results showed that YTHDC1 could bind to probe m^{6}A, but not to the non-methylated probe A (Figure 6H). On the other hand, more ESRP2 protein was shown to bind to probe A than probe m^{6}A (Figure 6H), suggesting that m^{6}A inhibits ESRP2 binding to Lnc-LSG1. These results confirm that YTHDC1 competitively inhibits ESRP2 binding to Lnc-LSG1 in an m^{6}A-dependent manner.

Furthermore, we investigated the effect of YTHDC1 on ESRP2 expression. As shown in Figure 6I, ESRP2 protein expression can be upregulated by overexpression of YTHDC1 and downregulated by knockdown of YTHDC1. However, ESRP2 mRNA expression was not regulated by YTHDC1 (Figure S4D). In addition, YTHDC1 could partially reverse the inhibitory effect of Lnc-LSG1 on ESRP2 protein without altering Lnc-LSG1 expression (Figures 6J and S4D). Moreover, siYTHDC1 can block the effect of METTL14 overexpression on ESRP2 expression and ubiquitin modification (Figures 6K and 6L). Taken together, these results suggest that METTL14 can inhibit ESRP2 and Lnc-LSG1 interaction by increased binding of YTHDC1 to the m^{6}A site in Lnc-LSG1.

DISCUSSION

m^{6}A methylation is the most prevalent post-transcriptional RNA modification in eukaryotic cells. It is reversible and catalyzed by corresponding enzymes, namely “writers,” “erasers,” and “readers.” Numerous studies have explored the dysregulation and specific functions of m^{6}A enzymes in various diseases and malignancies.[37,38] In this study, bioinformatics analysis was used to determine the role of m^{6}A modification in ccRCC progression. Using RNA sequencing and CNV data of ccRCC patients from TCGA database, Zhou et al. reported that genetic alterations of m^{6}A regulators in ccRCC were associated with decreased m^{6}A levels and poorer OS and DFS.[11] Zhong et al. clustered ccRCC patients into three m^{6}A modification patterns with distinct immune landscapes and prognoses. They reported that the m^{6}A gene signature was an independent prognostic factor for ccRCC.[39] In addition, Zhou et al.[11] and Gong et al.[40] found that METTL14 was downregulated in the TCGA KIRC cohort and was negatively associated with the ccRCC stage and OS. However, these findings were based on data obtained from online databases. Moreover, research based on *in vitro* and *in vivo* experiments is limited. Therefore, little information is currently available about the biological functions and molecular mechanisms of METTL14 in ccRCC progression.

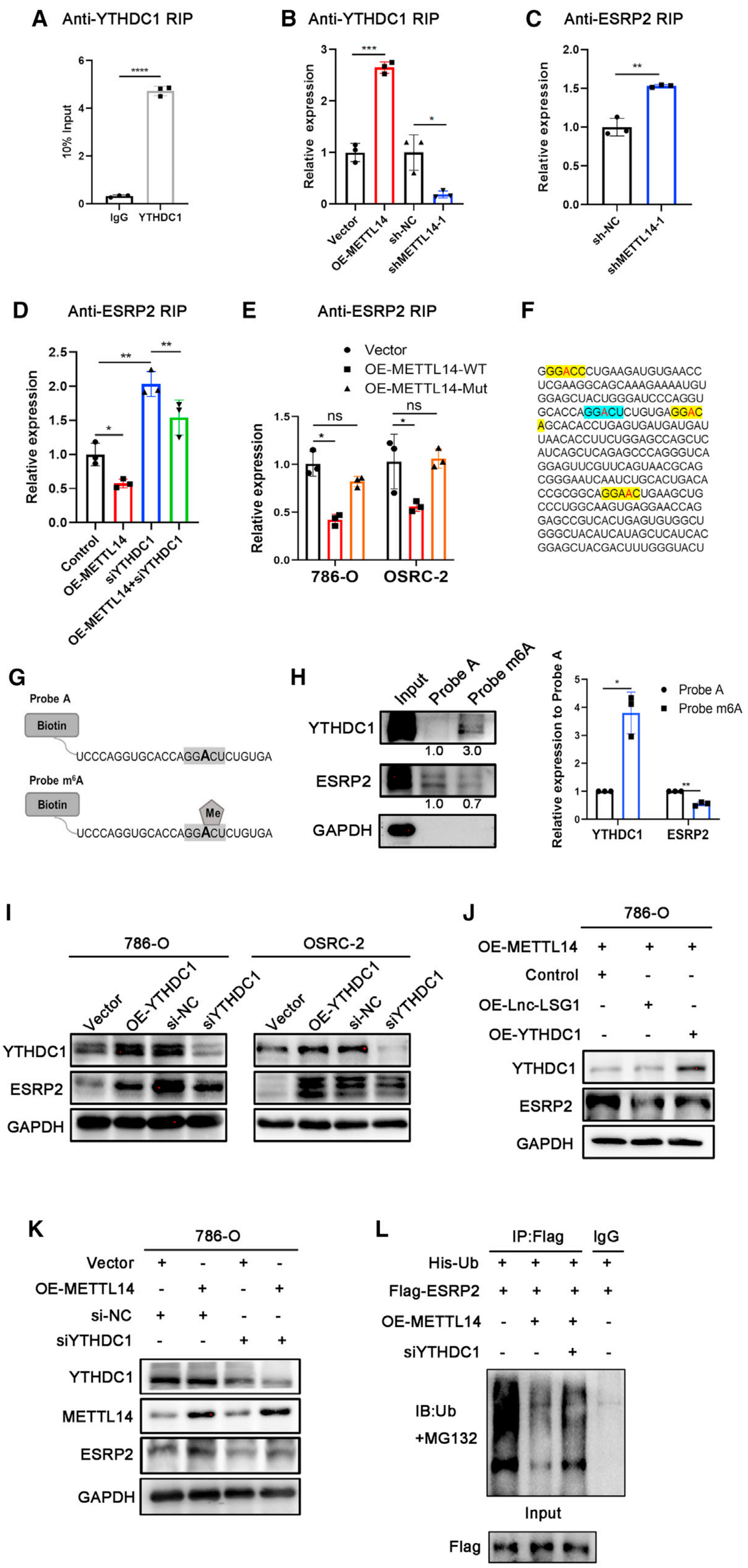

Figure 6. METTL14 inhibits ESRP2 and Lnc-LSG1 interaction through YTHDC1

(A and B) Anti-YTHDC1 RIP assay showed that Lnc-LSG1 could significantly bind to YTHDC1. RIP, RNA immunoprecipitation (A). METTL14 can regulate the interaction between Lnc-LSG1 and YTHDC1 (B). (C and D) Anti-ESRP2 RIP assay showed increased interaction between Lnc-LSG1 and ESRP2 in shMETTTL14-1 cells and decreased interaction in OE-METTL14 cells (C). Knocking down YTHDC1 increased the binding between Lnc-LSG1 and ESRP2 (D), and blocked the inhibitory effect of OE-METTL14 on the interaction between Lnc-LSG1 and ESRP2 (D). (E). Anti-ESRP2 RIP assay showed that METTL14-WT, but not METTL14-R298P, could inhibit the interaction between ESRP2 and Lnc-LSG1. (F) Four RRACH motifs in the region 0–300 nt of Lnc-LSG1. (G) Schematic drawing of probe A and probe m^6A. (H) RNA pull-down assay using probe A or probe m^6A, followed by western blotting assay for ESRP2, YTHDC1, and GAPDH protein. (I) Western blotting assay was performed to investigate the effect of YTHDC1 on ESRP2 protein expression. (J) Western blotting assay showed that siYTHDC1 could partly reverse the Lnc-LSG1 overexpression-induced inhibition on ESRP2 expression. (K and L) siYTHDC1 partly blocked the effect of METTL14 overexpression on ESRP2 expression (K) and ubiquitination levels (L). Data are presented as the mean ± SD of at least three independent experiments.

In this study, the decreased expression of METTL14 was verified in samples obtained from ccRCC patients in the SRRSH cohort and tissue microarrays. Furthermore, the results revealed that low METTL14 expression was correlated with poorer OS. In addition, METTL14 significantly inhibited the ccRCC migration and invasion *in vitro* and *in vivo*, suggesting that METTL14 has a tumor-suppressor role.

M^6A modification has also been identified in lncRNAs. Recently, several studies have shown that m^6A modification in lncRNAs is implicated in various cancers. For instance, METTL3- and YTHDF3-mediated m^6A methylation was shown to enhance the stability of MALAT1 in non-small cell lung cancer (NSCLC). MALAT1 functions as an endogenous RNA (ceRNA) sponging miR-1914-3p, thereby increasing expression of YAP, resulting in NSCLC metastasis and cisplatin resistance.[15] In this study, MeRIP-seq was used to explore the functions of m^6A-modified lncRNAs in ccRCC for the first time. The results revealed that Lnc-LSG1 was a downstream target of METTL14 with an anti-metastastic effect on ccRCC cells. Furthermore, METTL14 was shown to inhibit binding between Lnc-LSG1 and ESRP2 protein by recruiting YTHDC1 to a GGACU motif on Lnc-LSG1, eventually increasing the stability of ESRP2 protein.

LncRNAs have been shown to regulate protein stability through RNA-protein interactions.[41,42] In this study, the RNA pull-down and RIP assays showed a direct protein interaction between ESRP2 and Lnc-LSG1. The results revealed that Lnc-LSG1 inhibited ESRP2 protein expression by shortening its half-life, suggesting that the interaction between Lnc-LSG1 and ESRP2 could regulate ESRP2 expression through protein degradation. Treatment with MG132 can significantly increase ESRP2 protein levels. Therefore, we investigated the functional significance of Lnc-LSG1 in the ubiquitination of ESRP2 protein. Lnc-LSG1 was shown to increase ESRP2 ubiquitination levels, implying that Lnc-LSG1 promotes ESRP2 degradation via the ubiquitination pathway. Epithelial splicing regulatory proteins (ESRP1 and ESRP2) are splicing regulators expressed in epithelial cells. According to Mizutani et al., ESRP2 expression, but not ESRP1, was maintained in ccRCC. Furthermore, they showed that the knockdown of ESRP2 promoted OSRC-2 migration.[28] Our data confirmed the function of ESRP2 in ccRCC cells.

This study shows that Lnc-LSG1 promoted ccRCC metastasis via binding the ESRP2 protein, further facilitating ubiquitination and degradation. According to Mizutani et al., Arkadia ubiquitinates lysine residues 27 (Lys27) on ESRP2, suppressing cell proliferation. However, the knockdown of Arkadia failed to show any effect on cell migration. Furthermore, ESRP2 ubiquitination by Arkadia did not induce ESRP2 degradation,[28] suggesting that ubiquitination on Lys27 by Arkadia was not responsible for ESRP2 protein degradation. Based on the fact that Lnc-LSG1-induced ESRP2 ubiquitination promotes ESRP2 degradation and enhances ccRCC migration, we hypothesized that Arkadia and Lys27 of ESRP2 do not mediate the Lnc-LSG1 function. Further studies are required to reveal the specific E3-ligase and lysine residues that mediate the ESRP2 polyubiquitination induced by Lnc-LSG1.

Zhao et al. reported a tissue-specific isoform switch of fibroblast growth factor receptor 2 (FGFR2) from FGFR2 IIIb (epithelial isoform) to FGFR2 IIIc (mesenchymal isoform) in nearly 90% of ccRCC patients. FGFR2 IIIc ccRCC were larger in size with worse clinical outcomes compared with FGFR2 IIIb ccRCC.[43] ESRP2 can induce a substantial switch from FGFR2 IIIc to FGFR2 IIIb.[27] Lnc-LSG1 was shown to increase FGFR2 IIIc expression and decrease FGFR2 IIIb expression using a qRT-PCR assay. In contrast, METTL14 was shown to decrease FGFR2 IIIc expression and increase FGFR2 IIIb expression (Figure S4E). This is consistent with our finding that METTL14 promotes ESRP2 expression via Lnc-LSG1. Therefore, FGFR2 IIIb/IIIc was hypothesized to be a downstream target of ESRP2. However, the molecular mechanism of FGFR2 IIIb/IIIc involvement in ccRCC progression warrants further studies.

YTHDC1 is responsible for RNA splicing by recognizing m^6A methylation in RNAs.[44,45] Recently, Yoneda et al. proposed a competitive effect of YTHDC1 on RNA-protein interplay, and demonstrated that YTHDC1 can inhibit TLS protein binding to lncRNA pncRNA-D by m^6A modification.[32] In this study, an RNA pull-down assay with m^6A-modified and non-m^6A-modified RNA probes showed that m^6A methylation increases the binding of YTHDC1 to Lnc-LSG1. In contrast, m^6A methylation decreases the binding of ESRP2 to Lnc-LSG1, implying that YTHDC1 competitively blocks the interaction between ESRP2 and Lnc-LSG1 by binding to m^6A sites. In addition, the RIP assay showed that siYTHDC1 could disrupt the METTL14-induced inhibition on the interaction between Lnc-LSG1 and ESRP2. Furthermore, mutated METLL14 (METTL14-R298P) did not affect ESRP2 expression and ESRP2/Lnc-LSG1 interaction. Knocking down YTHDC1 can partially reverse the effect of wild-type METTL14 on ESRP2 expression and ubiquitination. Taken together, these results reveal that YTHDC1, an m^6A reader, is a mediator of METTL14 in inhibiting the interaction between Lnc-LSG1 and ESRP2.

In conclusion, we comprehensively elucidate the clinical relevance, functional roles, and detailed molecular mechanisms of METTL14 in ccRCC progression. For the first time, we provide insights into the function and mechanism of m^6A-modified lncRNA in ccRCC and identify a "METTL14-YTHDC1-Lnc-LSG1" regulation axis in ccRCC progression. Thus, this study highlights the vital roles of METTL14 and lncRNA m^6A modification in ccRCC development and may pave the way for developing novel biomarkers and therapies in ccRCC.

METHODS

Human samples, cell lines, and antibodies

The ccRCC specimens and matched adjacent normal tissues were obtained from 50 patients who underwent curative surgical resections from 2018 to 2020 in the Department of Urology, SRRSH, Zhejiang University School of Medicine, Hangzhou, China. The protocols for

tissue sample collection were approved by the Ethics Committee of SRRSH (IRB no. 20180226-70). Informed consent was obtained from all patients. The clinical data of patients are provided in Tables S1 and S2. The tissue microarray was purchased from Shanghai Liao Ding Biotechnology (Shanghai, China). The clinical data of patients are provided in Table S3. Three ccRCC cell lines, 786-O (established from a 58-year-old white male patient), Caki-1 (established from a 49-year-old white male patient), and OSRC-2 (established from a 52-year-old Japanese male patient) were purchased from the Cell Bank of Type Culture Collection of the Chinese Academy of Sciences. The 786-O and OSRC-2 cell lines were cultured in RPMI-1640 with 10% FBS (Cellmax, Peking, China), and the Caki-1 cell line was grown in McCoy 5A medium containing 10% FBS (Cellmax). All cell lines were incubated at 37°C under 5% CO_2 atmosphere.

The antibodies used in this study were: anti-METTL14 (rabbit polyclonal, HPA038002, Sigma-Aldrich), anti-m^6A (rabbit monoclonal, ab190886, Abcam), anti-ESRP2 (rabbit polyclonal, GTX31826, GeneTex; rabbit polyclonal, NBP2-13972, Novus), anti-Flag (mouse monoclonal, ab18230, Abcam), anti-Ubiquitin (rabbit monoclonal, ab134953, Abcam), anti-GAPDH (mouse monoclonal, ab8245, Abcam), anti-YTHDC1 (rabbit polyclonal, 14392-1-AP, Proteintech), and anti-IgG (rabbit polyclonal, Cell Signaling Technology, no. 2729).

Construction of smart silencer, small interfering RNAs (siRNAs), and plasmids: Transfection, lentivirus infection

The smart silencer targeting Lnc-LSG1, as well as siRNAs targeting ESRP2 and YTHDC1, were synthesized by RiboBio (Guangzhou, China); siRNA sequences are listed in Table S4. The smart silencer and siRNAs were transfected into cells using Lipofectamine RNAiMAX transfection reagent (Invitrogen, Carlsbad, CA, USA) according to the manufacturer's guidance. Ectopic expression plasmids of indicated genes were synthesized by GENECHEM (Shanghai, China) and transfected using Lipofectamine 3000 (Invitrogen). The METTL14-overexpressing and -knockdown lentivirus was designed, synthesized and collected by GENECHEM (Shanghai, China) and used to infect ccRCC cells with 8 mg/mL polybrene for 3 days. Stable infected cell lines were selected using puromycin (Selleck, Shanghai, China).

Bioinformatic analysis

The UALCAN (http://ualcan.path.uab.edu) online database was used to analyze the expression of METTL14 in normal and tumor tissues and in different ccRCC stages, grades, and metastasis status. GEPIA (http://gepia.cancer-pku.cn/) was used to investigate the prognostic role of METTL14. UALCAN and GEPIA tools deliver fast and customizable functionalities based on data from TCGA. They provide key interactive and customizable functions, including differential expression analysis, correlation analysis, and patient survival analysis.[46,47] The online tool catRAPID (http://s.tartaglialab.com/page/catrapid_group) was used to predict the proteins that could interact with Lnc-LSG1.[26] The CPC 2.0 (http://cpc2.gao-lab.org/index.php) was used to predict the protein-coding ability of Lnc-LSG1.[22]

CCK8 assay

The Cell Counting Kit-8 (Yeason, Hangzhou, China) was used to measured cell proliferation. In brief, 2×10^3 ccRCC cells per well were seeded onto 96-well plates and 10 μL CCK-8 solution was added at days 1, 2, 3, and 4. After incubation at 37°C for 2 h, absorbance for each well was measured at 450 nm.

Transwell assay

Migration and invasion assays were conducted using 8-μm pore filters (Millipore, Germany) coated with or without Matrigel (BD Biosciences, San Jose, CA, USA). The ccRCC cells were seeded into the top chamber with serum-free RPMI 1640 or McCoy 5A, and 10% FBS medium was added to the lower chamber. After 24 h of incubation, non-migrating or non-invasive cells on the upper chamber were gently wiped off, and cells on the membrane bottom were fixed with 4% paraformaldehyde for 15 min. After fixation, cells were further stained with crystal violet for 20 min and counted in three randomly chosen fields.

Wound healing assay

The ccRCC cells were plated in 6-well plates and cultured to 90% confluency. The cell monolayer was scratched using a 1-mL pipette tip, washed twice with PBS, and cultured in 1% FBS medium. Images of wound closure in three random fields were captured after 0, 24, and 48 h incubation and further analyzed by Image J software.

IHC

All samples were paraffin embedded and cut into 4 μm sections. After deparaffinization, rehydration with alcohol, and antigen retrieval using sodium citrate buffer, the tissue sections were treated with 3% H_2O_2 and blocked in 3% goat serum. Subsequently, the sections were incubated with METTL14 or ESRP2 antibodies at 4°C overnight, followed by incubation at room temperature for 1 h with secondary antibodies. The operator and the pathologists were blinded to the clinical and prognostic information of the patients.

The IHC score was calculated by multiplying the different staining intensities in three levels (weak = 1, medium = 2, strong = 3) with the percentage of ccRCC cells with positive staining (0%–25% = 1, 26%–50% = 2, 51%–75% = 3, 76%–100% = 4).

RNA extraction and qRT-PCR

Total RNA was isolated with TRIzol (Cwbiotech, Peking, China) according to the manufacturer's instructions. A total of 1 μg RNA was used for cDNA synthesis by the All-in-One cDNA Synthesis SuperMix for PCR (Bimake, Shanghai, China). The qRT-PCR reactions were performed using a 2× SYBR Green qPCR master mix (Bimake). The primer sequences are listed in Table S5.

Western blotting

For this assay, ccRCC cells or human samples were lysed using RIPA buffer (Beyotime, Shanghai, China) for 15 min on ice and then centrifuged at 12,000 × *g* for 20 min at 4°C. The supernatants were

collected and added to 5× loading buffer (Fdbio, Hangzhou, China). Proteins were further resolved in 10% SDS-PAGE, transferred onto a PVDF membrane (Bio-Rad, Hercules, CA, USA) and blocked in 5% nonfat milk. The membranes were immunoblotted with appropriate primary and secondary antibodies, and an enhanced chemiluminescence kit (Fdbio) was used to visualize specific protein bands.

RNA pull-down assay

The biotinylated probes were synthesized by Ribio (Guangzhou). Cells were lysed using IP buffer supplemented with RNase Inhibitor (Thermo Fisher Scientific, MA, USA) and protease inhibitor cocktail (Fdbio) at 4°C for 15 min. After centrifugation, the supernatants were collected and incubated with biotin-labeled RNA probes at 4°C overnight, followed by incubation with 20 μL streptavidin agarose beads for another 1 h at 4°C. Beads were collected and washed 10 times with IP buffer. Proteins retrieved by biotinylated RNA were analyzed by western blotting assay.

RNA immunoprecipitation assay

The RNA immunoprecipitation (RIP) experiments were performed using the Magna RIP RNA-Binding Protein Immunoprecipitation Kit (Millipore, MA, USA) according to the manufacturer's instructions. In brief, cells were lysed using RIP lysis buffer and incubated with the indicated antibodies and protein G magnetic beads, followed by protein digestion with proteinase K. The purified total RNA was subsequently subjected to qRT-PCR analysis.

MeRIP-seq assays

For MeRIP-seq, total RNA was isolated using TRIzol reagent. The obtained mRNA was further purified using the Dynabeads mRNA DIRECT Kit (Thermo Fisher) and fragmented by sonication. MeRIP-seq and library preparation were performed as per the reported protocol[33] with some modifications. In brief, sonicated mRNA was mixed with m^6A antibody (Synaptic Systems, 202003) in IP buffer and incubated under head-to-tail mixing at 4°C for 2 h. The mixture was supplemented with protein A magnetic beads (Thermo Fisher) and incubated under head-to-tail mixing at 4°C for another 2 h. The beads were then washed with IP buffer three times before elution with m^6A elution buffer two times. The eluates were combined and purified by an RNA Clean and Concentrator (Zymo, Orange, CA). The purified mRNA fragments were used to construct libraries with the TruSeq Stranded mRNA Library Prep Kit (Illumina, San Diego, CA). Sequencing was carried out on the Illumina HiSeq 2000 system with pair-end 150-bp read length. Reads were aligned to human genome version 38 (GRCh38) with TopHat. The longest isoform was retained if a gene had more than one isoform. Differential m^6A-modified peaks between IP and input samples were identified using exomePeak ($p < 0.01$).

Measurement of total m^6A mRNA levels

Total RNA was isolated using TRIzol reagent (Cwbiotech). The m^6A level was measured using an m^6A RNA methylation quantification kit (EpiGentek), according to the manufacturer's instructions.

FISH

The FISH probe labeled with Cy3 at the 5′ of Lnc-LSG1 was purchased from RiboBio (Guangzhou, China). The subcellular localization of Lnc-LSG1 was further investigated using a FISH Kit (Ribobio) as recommended by the manufacturer. A confocal laser scanning microscope (Nikon, Tokyo, Japan) was used to capture the images.

Animal experiments

All animal studies were conducted in accordance with the institutional guidelines approved by the Animal Research Ethics Committee of Zhejiang University.

For the xenograft tumor model, approximately 1×10^6 ccRCC cells suspended in 100 μL PBS were subcutaneously inoculated in the right flank of 5-week-old BALB/c nude mice. After 4 weeks, the xenograft tumors were collected and tumor volume was calculated according to the following formula: volume $=$ (width^2 $\times$ length)/2.

For the ccRCC orthotopic implantation model, approximately 1×10^6 ccRCC cells suspended in 30 μL Matrigel were injected under the renal capsule of 5-week-old BALB/c nude mice. After 6 weeks, the anesthetized mice were intraperitoneally injected with D-luciferin (Yeason) and imaged using an *in vivo* imaging system to detect tumor growth and metastasis. The mice were then sacrificed, and the lung, liver, spleen, and intestine tissues were harvested, imaged, and subjected to IHC staining and H&E staining.

For the lung metastasis model, approximately 5×10^5 ccRCC cells suspended in PBS were injected into the tail vein of 5-week-old mice. After 6–8 weeks, mice were anesthetized and lung metastasis was imaged as above. Lung tissues were further harvested, imaged, and subjected to H&E staining.

Statistical analysis

The statistical analysis of all experimental data was performed by GraphPad Prism 8.0. The survival curve of METTL14 was determined using the Kaplan-Meier method. All experiments were repeated more than three times. Statistical significance was considered as $^{*}p < 0.05$, $^{**}p < 0.01$, $^{***}p < 0.001$, and $^{****}p < 0.0001$.

SUPPLEMENTAL INFORMATION

Supplemental information can be found online at https://doi.org/10.1016/j.omtn.2021.12.024.

ACKNOWLEDGMENTS

This work was supported by the National Natural Science Foundation of China (grant nos. 82072809 and 81870484); the Joint construction project of Zhejiang Province and Ministry (grant no. 2020388200); the Key R & D plan of Zhejiang Province (grant no. 2019C03089); the Zhejiang Provincial Natural Science Foundation of China (grant no. LQ19H050011); the Zhejiang Medical and Health Plan Project (grant nos. 2018KY470, 2020KY595, 2019KY414, and 2019ZD027).

AUTHOR CONTRIBUTIONS

G.L., L.X., D.S., and L.D. contributed to conceiving the project and designing the research. D.S., L.D., Z.L., R.W., C.Y., and H.W were responsible for the most of *in vitro* and *in vivo* experiments. X.W., W.X., H.Y., and L.X. performed part of *in vitro* experiments and data analysis. D.S., M.W., and S.Y. wrote the manuscript. S.Y. and S.Z. participated in data analysis. All authors have read and approved the final manuscript.

DECLARATION OF INTERESTS

The authors declare no competing interests.

REFERENCES

1. Siegel, R.L., Miller, K.D., and Jemal, A. (2019). Cancer statistics, 2019. CA Cancer J. Clin. *69*, 7–34.
2. McDermott, D.F., Regan, M.M., Clark, J.I., Flaherty, L.E., Weiss, G.R., Logan, T.F., Kirkwood, J.M., Gordon, M.S., Sosman, J.A., Ernstoff, M.S., et al. (2005). Randomized phase III trial of high-dose interleukin-2 versus subcutaneous interleukin-2 and interferon in patients with metastatic renal cell carcinoma. J. Clin. Oncol. *23*, 133–141.
3. Esteller, M., and Pandolfi, P.P. (2017). The epitranscriptome of noncoding RNAs in cancer. Cancer Discov. *7*, 359–368.
4. Roundtree, I.A., and He, C. (2016). Nuclear m(6)A reader YTHDC1 regulates mRNA splicing. Trends Genet. *32*, 320.
5. Meyer, K.D., Patil, D.P., Zhou, J., Zinoviev, A., Skabkin, M.A., Elemento, O., Pestova, T.V., Qian, S.B., and Jaffrey, S.R. (2015). 5' UTR m(6)A promotes Cap-independent translation. Cell *163*, 999–1010.
6. Edens, B.M., Vissers, C., Su, J., Arumugam, S., Xu, Z.F., Shi, H., Miller, N., Ringeling, F.R., Ming, G.L., He, C., et al. (2019). FMRP modulates neural differentiation through m(6)A-dependent mRNA nuclear export. Cell Rep. *28*, 845.
7. Yang, X., Zhang, S., He, C., Xue, P., Zhang, L., He, Z., Zang, L., Feng, B., Sun, J., and Zheng, M. (2020). METTL14 suppresses proliferation and metastasis of colorectal cancer by down-regulating oncogenic long non-coding RNA XIST. Mol. Cancer *19*, 46.
8. Shi, H., Wei, J., and He, C. (2019). Where, when, and how: context-dependent functions of RNA methylation writers, readers, and erasers. Mol. Cell *74*, 640–650.
9. Shen, C., Sheng, Y., Zhu, A.C., Robinson, S., Jiang, X., Dong, L., Chen, H.Y., Su, R., Yin, Z., Li, W., et al. (2020). RNA demethylase ALKBH5 selectively promotes tumorigenesis and cancer stem cell self-renewal in acute myeloid leukemia. Cell Stem Cell *27*, 64.
10. Chen, X.X., Xu, M., Xu, X.N., Zeng, K.X., Liu, X.X., Pan, B., Li, C.M., Sun, L., Qin, J., Xu, T., et al. (2020). METTL14-mediated N6-methyladenosine modification of SOX4 mRNA inhibits tumor metastasis in colorectal cancer. Mol. Cancer *19*, 106.
11. Zhou, J., Wang, J., Hong, B., Ma, K., Xie, H., Li, L., Zhang, K., Zhou, B., Cai, L., and Gong, K. (2019). Gene signatures and prognostic values of m6A regulators in clear cell renal cell carcinoma - a retrospective study using TCGA database. Aging *11*, 1633–1647.
12. Chen, Y., Lin, Y., Shu, Y., He, J., and Gao, W. (2020). Interaction between N(6)-methyladenosine (m(6)A) modification and noncoding RNAs in cancer. Mol. Cancer *19*, 94.
13. Yi, Y.C., Chen, X.Y., Zhang, J., and Zhu, J.S. (2020). Novel insights into the interplay between m(6)A modification and noncoding RNAs in cancer. Mol. Cancer *19*, 121.
14. Meyer, K.D., Saletore, Y., Zumbo, P., Elemento, O., Mason, C.E., and Jaffrey, S.R. (2012). Comprehensive analysis of mRNA methylation reveals enrichment in 3' UTRs and near stop codons. Cell *149*, 1635–1646.
15. Jin, D., Guo, J., Wu, Y., Du, J., Yang, L., Wang, X., Di, W., Hu, B., An, J., Kong, L., et al. (2019). m(6)A mRNA methylation initiated by METTL3 directly promotes YAP translation and increases YAP activity by regulating the MALAT1-miR-1914-3p-YAP axis to induce NSCLC drug resistance and metastasis. J. Hematol. Oncol. *12*, 135.
16. Wen, S., Wei, Y., Zen, C., Xiong, W., Niu, Y., and Zhao, Y. (2020). Long non-coding RNA NEAT1 promotes bone metastasis of prostate cancer through N6-methyladenosine. Mol. Cancer *19*, 171.
17. Wang, P., Doxtader, K.A., and Nam, Y. (2016). Structural basis for cooperative function of Mettl3 and Mettl14 methyltransferases. Mol. Cell *63*, 306–317.
18. Wang, X., Feng, J., Xue, Y., Guan, Z., Zhang, D., Liu, Z., Gong, Z., Wang, Q., Huang, J., Tang, C., et al. (2016). Structural basis of N(6)-adenosine methylation by the METTL3-METTL14 complex. Nature *534*, 575–578.
19. Wang, W., Hu, W., Wang, Y., An, Y., Song, L., Shang, P., and Yue, Z. (2020). Long non-coding RNA UCA1 promotes malignant phenotypes of renal cancer cells by modulating the miR-182-5p/DLL4 axis as a ceRNA. Mol. Cancer *19*, 18.
20. Hirata, H., Hinoda, Y., Shahryari, V., Deng, G., Nakajima, K., Tabatabai, Z.L., Ishii, N., and Dahiya, R. (2015). Long noncoding RNA MALAT1 promotes aggressive renal cell carcinoma through Ezh2 and interacts with miR-205. Cancer Res. *75*, 1322–1331.
21. Li, J.K., Chen, C., Liu, J.Y., Shi, J.Z., Liu, S.P., Liu, B., Wu, D.S., Fang, Z.Y., Bao, Y., Jiang, M.M., et al. (2017). Long noncoding RNA MRCCAT1 promotes metastasis of clear cell renal cell carcinoma via inhibiting NPR3 and activating p38-MAPK signaling. Mol. Cancer *16*, 111.
22. Kang, Y.J., Yang, D.C., Kong, L., Hou, M., Meng, Y.Q., Wei, L., and Gao, G. (2017). CPC2: a fast and accurate coding potential calculator based on sequence intrinsic features. Nucleic Acids Res. *45*, W12–W16.
23. Joung, J., Engreitz, J.M., Konermann, S., Abudayyeh, O.O., Verdine, V.K., Aguet, F., Gootenberg, J.S., Sanjana, N.E., Wright, J.B., Fulco, C.P., et al. (2017). Genome-scale activation screen identifies a lncRNA locus regulating a gene neighbourhood. Nature *548*, 343–346.
24. Huarte, M. (2015). The emerging role of lncRNAs in cancer. Nat. Med. *21*, 1253–1261.
25. Ferre, F., Colantoni, A., and Helmer-Citterich, M. (2016). Revealing protein-lncRNA interaction. Brief. Bioinform. *17*, 106–116.
26. Bellucci, M., Agostini, F., Masin, M., and Tartaglia, G.G. (2011). Predicting protein associations with long noncoding RNAs. Nat. Methods *8*, 444–445.
27. Warzecha, C.C., Sato, T.K., Nabet, B., Hogenesch, J.B., and Carstens, R.P. (2009). ESRP1 and ESRP2 are epithelial cell-type-specific regulators of FGFR2 splicing. Mol. Cell *33*, 591–601.
28. Mizutani, A., Koinuma, D., Seimiya, H., and Miyazono, K. (2016). The Arkadia-ESRP2 axis suppresses tumor progression: analyses in clear-cell renal cell carcinoma. Oncogene *35*, 3514–3523.
29. Liu, B., Sun, L., Liu, Q., Gong, C., Yao, Y., Lv, X., Lin, L., Yao, H., Su, F., Li, D., et al. (2015). A cytoplasmic NF-kappaB interacting long noncoding RNA blocks IkappaB phosphorylation and suppresses breast cancer metastasis. Cancer Cell *27*, 370–381.
30. Zuo, X., Chen, Z., Gao, W., Zhang, Y., Wang, J., Wang, J., Cao, M., Cai, J., Wu, J., and Wang, X. (2020). M6A-mediated upregulation of LINC00958 increases lipogenesis and acts as a nanotherapeutic target in hepatocellular carcinoma. J. Hematol. Oncol. *13*, 5.
31. Ban, Y., Tan, P., Cai, J., Li, J., Hu, M., Zhou, Y., Mei, Y., Tan, Y., Li, X., Zeng, Z., et al. (2020). LNCAROD is stabilized by m6A methylation and promotes cancer progression via forming a ternary complex with HSPA1A and YBX1 in head and neck squamous cell carcinoma. Mol. Oncol. *14*, 1282–1296.
32. Yoneda, R., Ueda, N., Uranishi, K., Hirasaki, M., and Kurokawa, R. (2020). Long noncoding RNA pncRNA-D reduces cyclin D1 gene expression and arrests cell cycle through RNA m(6)A modification. J. Biol. Chem. *295*, 5626–5639.
33. Dominissini, D., Moshitch-Moshkovitz, S., Schwartz, S., Salmon-Divon, M., Ungar, L., Osenberg, S., Cesarkas, K., Jacob-Hirsch, J., Amariglio, N., Kupiec, M., et al. (2012). Topology of the human and mouse m6A RNA methylomes revealed by m6A-seq. Nature *485*, 201–206.
34. Chen, K., Lu, Z., Wang, X., Fu, Y., Luo, G.Z., Liu, N., Han, D., Dominissini, D., Dai, Q., Pan, T., et al. (2015). High-resolution N(6)-methyladenosine (m(6) A) map using photo-crosslinking-assisted m(6) A sequencing. Angew. Chem. Int. Ed. *54*, 1587–1590.
35. Visvanathan, A., and Somasundaram, K. (2018). mRNA traffic control reviewed: N6-methyladenosine (m(6) A) takes the driver's seat. Bioessays *40*. https://doi.org/10.1002/bies.201700093.

36. Sledz, P., and Jinek, M. (2016). Structural insights into the molecular mechanism of the m(6)A writer complex. eLife *5*, e18434.

37. Jiang, X., Liu, B., Nie, Z., Duan, L., Xiong, Q., Jin, Z., Yang, C., and Chen, Y. (2021). The role of m6A modification in the biological functions and diseases. Signal Transduct. Target. Ther. *6*, 74.

38. Huo, F.C., Zhu, Z.M., and Pei, D.S. (2020). N(6)-Methyladenosine (m(6) A) RNA modification in human cancer. Cell Prolif. *53*, e12921.

39. Zhong, J., Liu, Z., Cai, C., Duan, X., Deng, T., and Zeng, G. (2021). m(6)A modification patterns and tumor immune landscape in clear cell renal carcinoma. J. Immunother. Cancer *9*, e001646.

40. Gong, D., Zhang, J., Chen, Y., Xu, Y., Ma, J., Hu, G., Huang, Y., Zheng, J., Zhai, W., and Xue, W. (2019). The m(6)A-suppressed P2RX6 activation promotes renal cancer cells migration and invasion through ATP-induced Ca(2+) influx modulating ERK1/2 phosphorylation and MMP9 signaling pathway. J. Exp. Clin. Cancer Res. *38*, 233.

41. McHugh, C.A., Chen, C.K., Chow, A., Surka, C.F., Tran, C., McDonel, P., Pandya-Jones, A., Blanco, M., Burghard, C., Moradian, A., et al. (2015). The Xist lncRNA interacts directly with SHARP to silence transcription through HDAC3. Nature *521*, 232–236.

42. Gupta, R.A., Shah, N., Wang, K.C., Kim, J., Horlings, H.M., Wong, D.J., Tsai, M.C., Hung, T., Argani, P., Rinn, J.L., et al. (2010). Long non-coding RNA HOTAIR reprograms chromatin state to promote cancer metastasis. Nature *464*, 1071–1076.

43. Zhao, Q., Caballero, O.L., Davis, I.D., Jonasch, E., Tamboli, P., Yung, W.K., Weinstein, J.N., Kenna Shaw for TCGA Research Network, Strausberg, R.L., and Yao, J. (2013). Tumor-specific isoform switch of the fibroblast growth factor receptor 2 underlies the mesenchymal and malignant phenotypes of clear cell renal cell carcinomas. Clin. Cancer Res. *19*, 2460–2472.

44. Xiao, W., Adhikari, S., Dahal, U., Chen, Y.S., Hao, Y.J., Sun, B.F., Sun, H.Y., Li, A., Ping, X.L., Lai, W.Y., et al. (2016). Nuclear m(6)A reader YTHDC1 regulates mRNA splicing. Mol. Cell *61*, 507–519.

45. Zhang, Z., Theler, D., Kaminska, K.H., Hiller, M., de la Grange, P., Pudimat, R., Rafalska, I., Heinrich, B., Bujnicki, J.M., Allain, F.H., et al. (2010). The YTH domain is a novel RNA binding domain. J. Biol. Chem. *285*, 14701–14710.

46. Tang, Z., Li, C., Kang, B., Gao, G., Li, C., and Zhang, Z. (2017). GEPIA: a web server for cancer and normal gene expression profiling and interactive analyses. Nucleic Acids Res. *45*, W98–W102.

47. Chandrashekar, D.S., Bashel, B., Balasubramanya, S.A.H., Creighton, C.J., Ponce-Rodriguez, I., Chakravarthi, B., and Varambally, S. (2017). UALCAN: a portal for facilitating tumor subgroup gene expression and survival analyses. Neoplasia *19*, 649–658.

OMTN, Volume *27*

Supplemental information

METTL14-mediated Lnc-LSG1 m6A modification inhibits clear cell renal cell carcinoma metastasis via regulating ESRP2 ubiquitination

Danyang Shen, Lifeng Ding, Zeyi Lu, Ruyue Wang, Chenhao Yu, Huan Wang, Qiming Zheng, Xuliang Wang, Wanjiang Xu, Haifeng Yu, Liwei Xu, Mingchao Wang, Shicheng Yu, Shibin Zhu, Jun Qian, Liqun Xia, and Gonghui Li

a

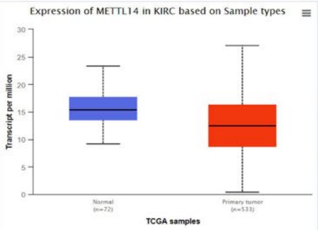

KIRC : Kidney renal clear cell carcinoma

Comparison	Statistical significance
Normal-vs-Primary	3.26019999885574E-09

b

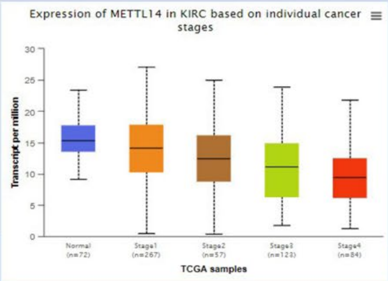

KIRC : Kidney renal clear cell carcinoma
Note

Hide statistics

Comparison	Statistical significance
Normal-vs-Stage1	1.479730E-03
Normal-vs-Stage2	2.835400E-03
Normal-vs-Stage3	2.6416646647931E-12
Normal-vs-Stage4	2.32702745961433E-13
Stage1-vs-Stage2	1.533420E-01
Stage1-vs-Stage3	1.60779999980321E-07
Stage1-vs-Stage4	1.89779999493211E-08
Stage2-vs-Stage3	6.635600E-02
Stage2-vs-Stage4	1.355810E-02
Stage3-vs-Stage4	2.789200E-01

c

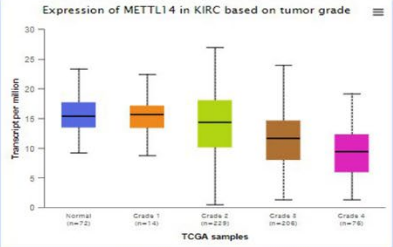

KIRC : Kidney renal clear cell carcinoma
Note

Subtype descriptions

Grade 1	Well differentiated (low grade)	Grade 2	Moderately differentiated (intermediate grade)
Grade 3	Poorly differentiated (high grade)	Grade 4	Undifferentiated (high grade)

Hide statistics

Comparison	Statistical significance
Normal-vs-Grade 1	5.663600E-01
Normal-vs-Grade 2	3.662700E-03
Normal-vs-Grade 3	2.17410534020246E-11
Normal-vs-Grade 4	1.62447832963153E-12
Grade 1-vs-Grade 2	1.233750E-01
Grade 1-vs-Grade 3	6.648900E-04
Grade 1-vs-Grade 4	1.96769999960544E-07
Grade 2-vs-Grade 3	3.91340000005513E-06
Grade 2-vs-Grade 4	5.06550357215474E-12
Grade 3-vs-Grade 4	4.408500E-04

d

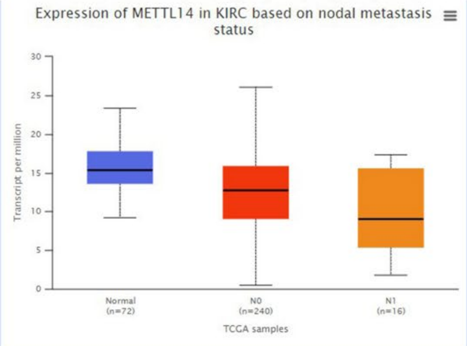

KIRC : Kidney renal clear cell carcinoma
Note

pathologic_N descriptions

N0	No regional lymph node metastasis	N1	Metastases in 1 to 3 axillary lymph nodes
N2	Metastases in 4 to 9 axillary lymph nodes	N3	Metastases in 10 or more axillary lymph nodes

Hide statistics

Comparison	Statistical significance
Normal-vs-N0	1.91839999996724E-07
Normal-vs-N1	3.98590000005861E-07
N0-vs-N1	3.567700E-02

e

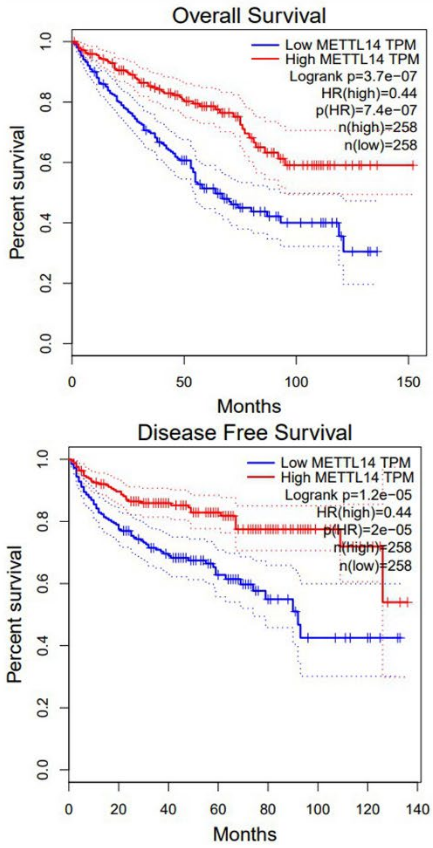

Figure S1. (a), (b), (c). According to the UAlCAN database, METTL14 was downregulated in the ccRCC primary tumor when compared to normal tissues (a). The higher the tumor stage (b) and grade (c), the lower the METTL14 expression. (d). METTL14 expression was lower in metastatic ccRCC tissues than that in non-metastatic tissues. (d). Kaplan-Meier analysis in the GEPIA database showed that lower METTL14 expression is associated with poorer OS and DFS. All these data were obtained on 03/16/2021. Data were presented as the mean ± SD of at least three independent experiments.

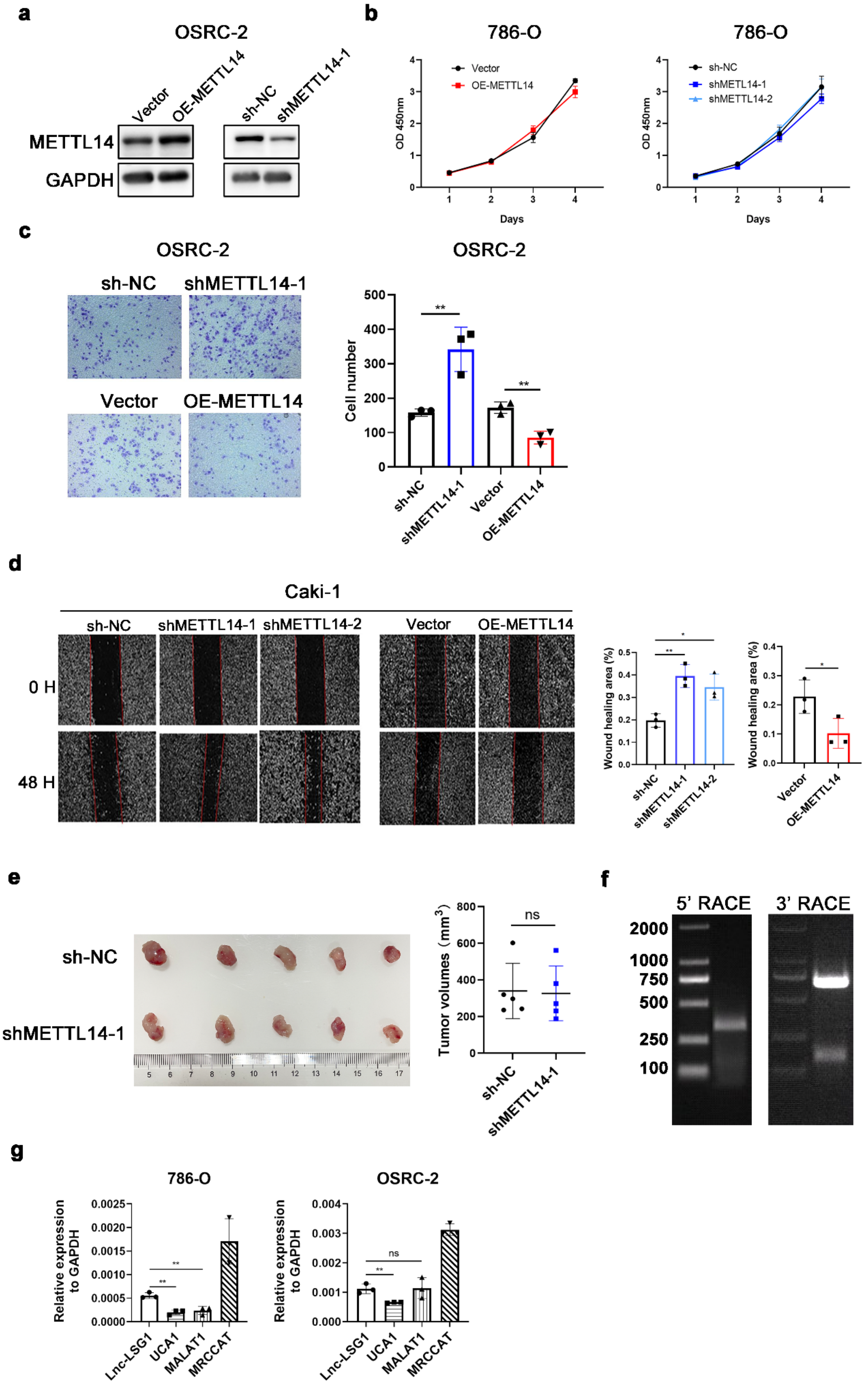
a
OSRC-2
Vector
OE-METTL14
sh-NC
shMETTL14-1
METTL14
GAPDH
b
786-O
Vector
OE-METTL14
OD 450nm
Days
786-O
sh-NC
shMETTL14-1
shMETTL14-2
OD 450nm
Days
c
OSRC-2
sh-NC
shMETTL14-1
Vector
OE-METTL14
OSRC-2
Cell number
d
Caki-1
sh-NC
shMETTL14-1
shMETTL14-2
Vector
OE-METTL14
0 H
48 H
Wound healing area (%)
e
sh-NC
shMETTL14-1
Tumor volumes（mm3）
ns
f
5' RACE
3' RACE
2000
1000
750
500
250
100
g
786-O
OSRC-2
Relative expression to GAPDH
Lnc-LSG1
UCA1
MALAT1
MRCCAT

h

ID	Label	Coding probability	Peptide length(aa)	Fickett score	Isoelectric point	ORF integrity
Lnc-LSG1	noncoding	0.0318156	64	0.3284	8.90765380859	complete

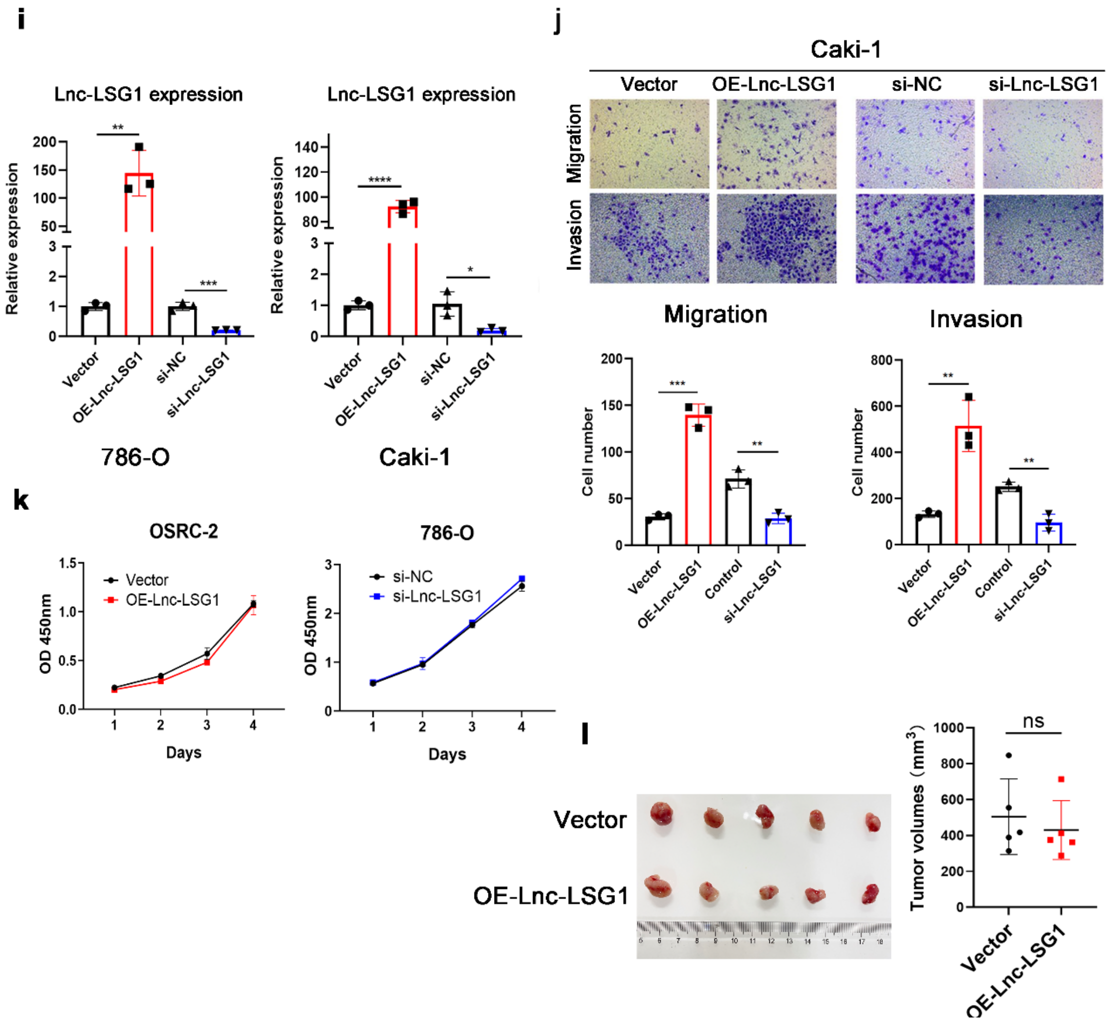

Figure S2 (a). METTL14 knockdown and overexpression were confirmed by western blot assay in OSRC-2 cells. (b). METTL14 cannot regulate 786-O proliferation. (c). Transwell assay showed that METTL14 inhibits OSRC-2 migration. (d). Wound healing assay were performed to detect the migratory and invasive abilities of Caki-1 cells after METTL14 depletion and overexpression. Magnification, 100×. (e). The mice were sacrificed and tumor volume was measured after 4 weeks OSRC-2 cells injection. Left panel: Image of the xenograft tumors from sh-NC and sh-METTL14-1 group. Right panel: Average tumor volume with SD (n = 5 per group). (f). 5′ and 3′

RACE assays in 786-O cells detected the whole sequence of Lnc-LSG1.The figure showed PCR products from the 5′-RACE and 3′-RACE assays. (g). The expression of Lnc-LSG1 and some highly expressed lncRNAs in ccRCC cells. (h). Protein coding potential of Lnc-LSG1-4:2 as predicted by CPC 2.0. (i). Lnc-LSG1 knockdown and overexpression were confirmed by RT-qPCR assay. (j). Transwell assay showed the regulatory effect of Lnc-LSG1 on Caki-1 migration and invasion. (k). CCK8 assay showed Lnc-LSG1 has no effect on cell proliferation. (l). The mice were sacrificed and tumor volume was measured after 4 weeks OSRC-2 cells injection. Left panel: Image of the xenograft tumors from Vector and OE-Lnc-LSG1 group. Right panel: Average tumor volume with SD (n = 5 per group). *P < 0.05, **P < 0.01, ***P < 0.001, ****P<0.0001. ns: no significance. Data were presented as the mean ± SD of at least three independent experiments.

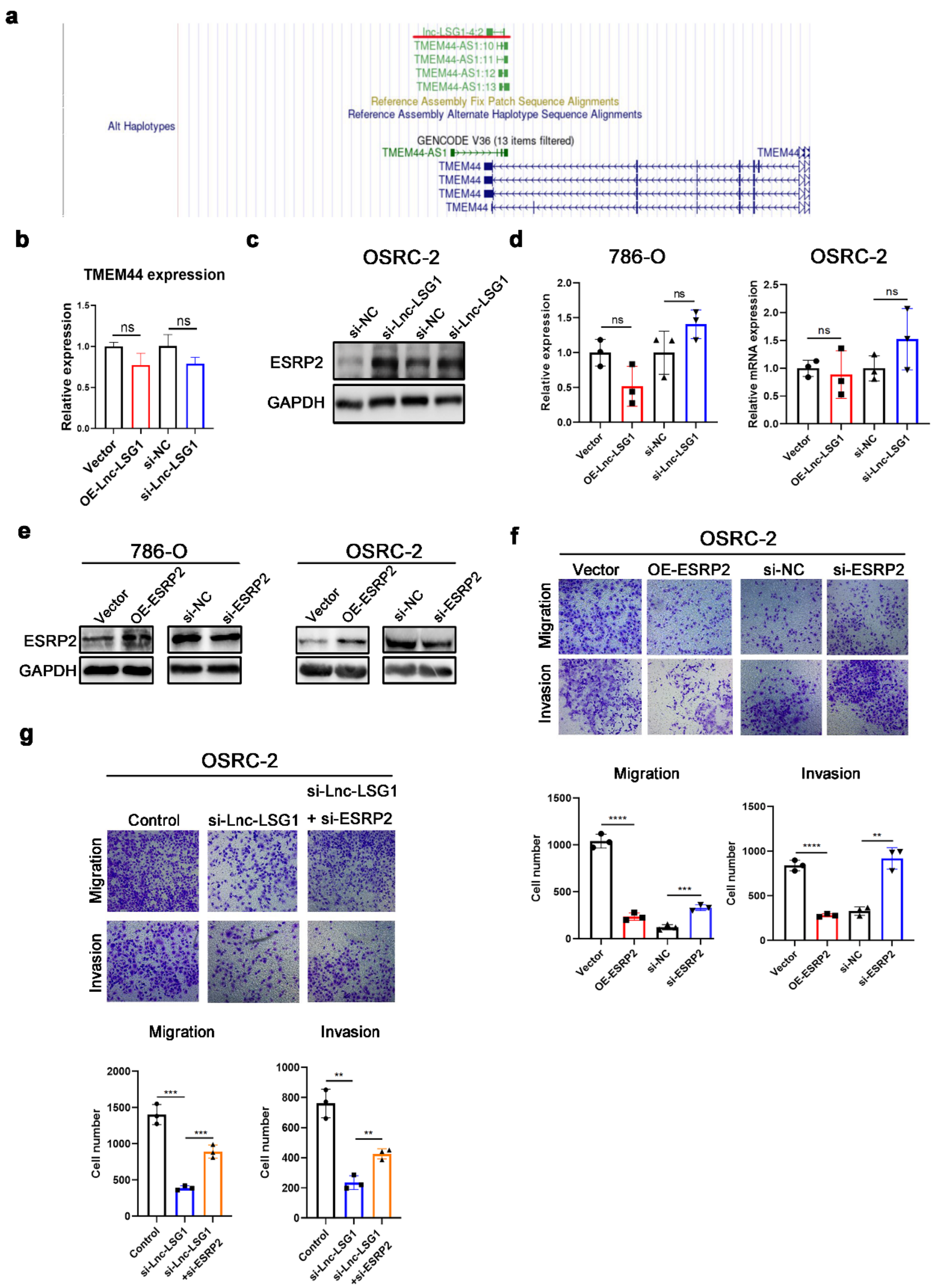

Figure S3. (a). The location of Lnc-LSG1-4:2 was retrieved by the UCSC (http://genome.ucsc.edu/) database. These data were obtained on 03/16/2021. (b). Lnc-LSG1 showed no effect on TMEM44 mRNA expression. (c). si-Lnc-LSG1 can increase ESPR2 protein level. (d). Lnc-LSG1 showed no effect on ESRP2 mRNA

expression. (e). Knockdown and overexpression ESRP2 were confirmed by western blot assay. (f). Transwell assay showed that ESRP2 can inhibit the migration and invasion of OSRC-2 cells. (g). si-ESRP2 can rescue the inhibited migratory and invasive ability of OSRC-2 cells induced by si-Lnc-LSG1. ns: no significance. *P < 0.05, **P < 0.01, ***P < 0.001, ****P<0.0001, ns: no significance. Data were presented as the mean ± SD of at least three independent experiments.

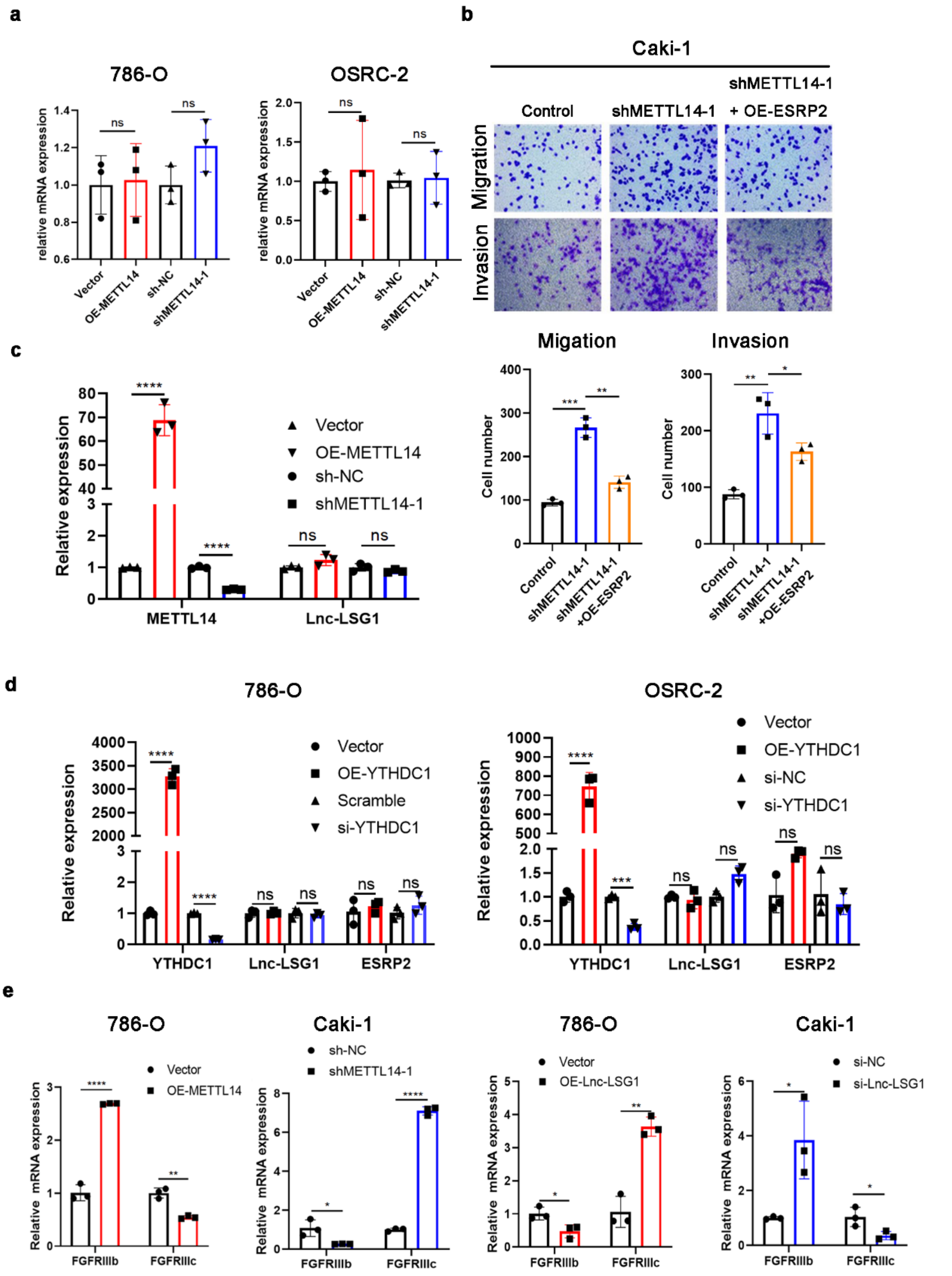

Figure S4 (a). RT-qPCR assay showed that METTL14 cannot regulate ESRP2 mRNA expression. (b). ESRP2 overexpression can reverse the enhanced migration and invasion of Caki-1 cells induced by shMETTL14-1. (c). METTL14 cannot regulate Lnc-LSG1 expression. (d). YTHDC1 cannot regulate Lnc-LSG1 and ESRP2 mRNA

expression. (e). RT-qPCR assay revealed the regulatory effect of METTL14 and Lnc-LSG1 on FGFR2 IIIb and FGFR2IIIc. *P < 0.05, **P < 0.01, ***P < 0.001, ****P<0.0001, ns: no significance. Data were presented as the mean ± SD of at least three independent experiments.

Table S1. Information of patients from Sir Run Run Shaw Hospital （RT-qPCR samples）									
NO	Gender	Age	Metastasis	Pathology	Stage	Grade	T	N	M
1	Male	48	no	ccRCC	I	2	1b	0	0
2	Male	56	no	ccRCC	III	3	3a	0	0
3	Male	84	no	ccRCC	I	2	1b	0	0
4	Male	54	no	ccRCC	II	3	2a	0	0
5	Male	81	no	ccRCC	I	3	1a	0	0
6	Male	76	no	ccRCC	I	3	1a	0	0
7	Male	50	no	ccRCC	I	2	1a	0	0
8	Male	71	no	ccRCC	I	3	1b	0	0
9	Male	59	no	ccRCC	I	2	1a	0	0
10	Male	55	no	ccRCC	II	3	2a	0	0
11	Female	75	no	ccRCC	I	2	1b	0	0
12	Male	72	no	ccRCC	I	3	1a	0	0
13	Female	73	no	ccRCC	I	2	1b	0	0
14	Male	60	no	ccRCC	I	3	1a	0	0
15	Male	37	no	ccRCC	I	2	1a	0	0
16	Male	70	no	ccRCC	I	3	1a	0	0
17	Male	41	no	ccRCC	II	2	2b	0	0
18	Male	57	no	ccRCC	I	3	1a	0	0
19	Male	60	no	ccRCC	I	3	1a	0	0
20	Female	45	no	ccRCC	II	3	2a	0	0
21	Male	42	no	ccRCC	I	3	1a	0	0
22	Male	54	no	ccRCC	I	2	1b	0	0
23	Male	49	no	ccRCC	I	2	1a	0	0
24	Female	56	no	ccRCC	II	2	2a	0	0
25	Female	70	no	ccRCC	I	2	1b	0	0
26	Female	61	no	ccRCC	I	2	1b	0	0
27	Male	69	no	ccRCC	I	2	1b	0	0
28	Male	32	no	ccRCC	I	2	1a	0	0
29	Male	81	no	ccRCC	I	2	1b	0	0
30	Female	52	no	ccRCC	I	2	1a	0	0
31	Male	79	no	ccRCC	I	3	1a	0	0
32	Male	31	no	ccRCC	I	2	1b	0	0
33	Female	85	no	ccRCC	I	2	1b	0	0
34	Male	56	no	ccRCC	I	2	1a	0	0
35	Male	46	no	ccRCC	I	2	1a	0	0
36	Female	55	no	ccRCC	I	2	1a	0	0
37	Male	70	no	ccRCC	I	2	1a	0	0
38	Female	79	no	ccRCC	I	2	1b	0	0
39	Male	63	no	ccRCC	I	2	1b	0	0
40	Male	56	no	ccRCC	I	2	1a	0	0
41	Male	37	no	ccRCC	I	2	1b	0	0
42	Male	72	no	ccRCC	I	2	1b	0	0
43	Male	40	no	ccRCC	I	2	1a	0	0
44	Male	64	no	ccRCC	II	2	2a	0	0
45	Female	52	no	ccRCC	I	2	1a	0	0
46	Male	58	no	ccRCC	I	2	1a	0	0
47	Male	57	no	ccRCC	I	2	1a	0	0
48	Male	60	no	ccRCC	I	3	1a	0	0
49	Female	73	no	ccRCC	I	2	1b	0	0
50	Female	51	no	ccRCC	I	2	1a	0	0

Table S2. Information of patients from Sir Run Run Shaw Hospital （IHC samples）										
NO	Gender	Age	Metastasis	Pathology	Stage	Grade	Tumor Size (cm)	T	N	M
1	M	60	no	ccRCC	II	I	8*5	T2a	N0	M0
2	F	52	no	ccRCC	I	IV	4.8*4.5	T1b	N0	M0
3	M	71	no	ccRCC	I	II	4*3*3	T1a	N0	M0
4	F	51	no	ccRCC	I	II	3*2.5*1.7	T1a	N0	M0
5	M	42	no	ccRCC	II	III	8.3*8*5.5	T2a	N0	M0
6	M	60	no	ccRCC	I	II	4*3.7	T1a	N0	M0
7	F	60	no	ccRCC	I	II	3.5*3.5	T1a	N0	M0
8	M	62	no	ccRCC	I	III	2.3*2.2	T1a	N0	M0
9	F	70	no	ccRCC	I	III	3.8*3.5	T1a	N0	M0
10	M	43	no	ccRCC	I	II	7*4.7	T1b	N0	M0
11	M	60	no	ccRCC	I	III	3*3	T1a	N0	M0
12	M	33	no	ccRCC	I	IV	5.7*4	T1b	N0	M0
13	M	52	no	ccRCC	I	II	1.8*1.6	T1a	N0	M0
14	M	51	no	ccRCC	I	II	3.5*3	T1a	N0	M0
15	F	50	no	ccRCC	I	I	1.2*1	T1a	N0	M0
16	M	59	no	ccRCC	I	II	2.2*1.8	T1a	N0	M0
17	M	79	no	ccRCC	I	III	4.5*4	T1b	N0	M0
18	M	50	no	ccRCC	I	III	5.1*3.4	T1b	N0	M0
19	F	67	no	ccRCC	I	II	2.2*1.6	T1a	N0	M0
20	F	73	no	ccRCC	I	III	7*5.8	T1b	N0	M0
21	M	43	no	ccRCC	I	I	3.7*3.5	T1a	N0	M0
22	M	44	no	ccRCC	I	II	3.8*3.5	T1a	N0	M0
23	M	40	no	ccRCC	I	III	2.6*2.2*1.7	T1a	N0	M0
24	F	55	no	ccRCC	I	I	1.2*1.2	T1a	N0	M0
25	F	28	no	ccRCC	I	II	6.2*5.8	T1b	N0	M0
26	M	56	no	ccRCC	I	II	6.3*5.1	T1b	N0	M0
27	M	60	no	ccRCC	I	II	2.7*2.4	T1a	N0	M0
28	M	70	no	ccRCC	I	III	1.7*1.6	T1a	N0	M0
29	F	57	no	ccRCC	I	II	6*4.5*3.5	T1b	N0	M0
30	M	54	no	ccRCC	I	II	5.3*3	T1b	N0	M0
31	M	34	no	ccRCC	I	I	1.2*1.2	T1a	N0	M0
32	F	74	no	ccRCC	I	II	5*3.2	T1b	N0	M0
33	M	43	no	ccRCC	I	II	2.6*1.8	T1a	N0	M0
34	M	45	no	ccRCC	I	II	1.8*1.8	T1a	N0	M0
35	M	64	no	ccRCC	I	II	3.2*2.4	T1a	N0	M0
36	F	46	no	ccRCC	II	II	7.2*6.5	T2a	N0	M0
37	F	51	no	ccRCC	I	II	3.3*3	T1a	N0	M0
38	M	54	no	ccRCC	I	II	2.5*2	T1a	N0	M0
39	M	66	no	ccRCC	II	IV	9*7.5	T2a	N0	M0
40	F	71	no	ccRCC	I	II	4.5*4	T1b	N0	M0

Table S3. Information of patients from tissue microarray

NO	Gender	Age	Metastasis	Pathology	Stage	Grade	Tumor Size (cm)	T	N	M	Survival time (month)	Status
1	F	52	no	ccRCC	I	II	6.5×6×5	T1b	N0	M0	15.4	Dead
2	M	56	no	ccRCC	III	I	3.5×3×3	T3a	N0	M0	97.8	Alive
3	F	73	no	ccRCC	I	I	5×5×4	T1b	N0	M0	59.20	Alive
4	M	55	no	ccRCC	I	II	5×4×4	T1b	N0	M0	97.34	Alive
5	M	59	no	ccRCC	I	II	5.5×5×5	T1b	N0	M0	97.34	Alive
6	M	69	no	ccRCC	I	II	4.5×4×4	T1b	N0	M0	97.11	Alive
7	M	72	no	ccRCC	II	I	10×6×5	T2a	N0	M0	93.9	Alive
8	M	59	no	ccRCC	II	II	15×9×6.5	T2b	N0	M0	7.2	Dead
9	M	63	no	ccRCC	I	II	7×5×3.5	T1b	N0	M0	95.93	Alive
10	F	52	no	ccRCC	I	II	7×7×6	T1b	N0	M0	95.64	Alive
11	M	67	no	ccRCC	I	I	2.8×2.5×2.5	T1a	N0	M0	95.51	Alive
12	M	66	no	ccRCC	II	II	8×8×7	T2a	N0	M0	95.41	Alive
13	F	57	no	ccRCC	II	II	8×7×5.5	T2a	N0	M0	56.1	Dead
14	M	57	no	ccRCC	III	IV	12×8.5×7	T3b	N0	M0	5.6	Dead
15	M	61	no	ccRCC	I	II	7×5×5	T1b	N0	M0	95.02	Alive
16	F	79	no	ccRCC	I	II	3×3×2.5	T1a	N0	M0	22.5	Dead
17	F	49	no	ccRCC	I	I	1.2×1×0.8	T1a	N0	M0	94.03	Alive
18	F	72	no	ccRCC	I	II	6×5.5×5	T1b	N0	M0	93.38	Alive
19	F	64	no	ccRCC	I	II	3×3×3	T1a	N0	M0	92.75	Alive
20	M	38	no	ccRCC	I	I	2.5×2×2	T1a	N0	M0	92.69	Alive
21	M	58	no	ccRCC	I	II	4.5×4×3	T1b	N0	M0	92.00	Alive
22	F	61	no	ccRCC	I	II	5×4.5×4	T1b	N0	M0	91.93	Alive
23	M	60	no	ccRCC	II	III	9×8×6	T2a	N0	M0	91.57	Alive
24	M	55	no	ccRCC	I	III	4×3.5×3.5	T1a	N0	M0	91.51	Alive
25	M	75	no	ccRCC	III	III	4×3.5×3	T3a	N0	M0	91.5	Alive
26	F	63	no	ccRCC	I	II	3.5×2.5×2	T1a	N0	M0	91.15	Alive
27	F	45	no	ccRCC	II	II	8×7×6	T2a	N0	M0	90.92	Alive
28	M	51	no	ccRCC	I	II	2×2×1.5	T1a	N0	M0	90.79	Alive
29	M	40	no	ccRCC	I	II	6×3.5×3.5	T1b	N0	M0	90.13	Alive
30	M	78	no	ccRCC	I	III	5×5×4.5	T1b	N0	M0	90.00	Alive
31	M	53	no	ccRCC	I	I	7×6×5	T1b	N0	M0	89.93	Alive
32	F	61	no	ccRCC	I	II	5×4×4	T1b	N0	M0	89.41	Alive
33	M	61	no	ccRCC	III	II	8×8×6	T3a	N0	M0	88.6	Alive
34	M	62	no	ccRCC	I	II	7×6×5	T1b	N0	M0	88.52	Alive
35	M	63	no	ccRCC	I	II	7×6×4	T1b	N0	M0	98.9	Alive
36	F	60	no	ccRCC	I	II	6×6×5	T1b	N0	M0	87.21	Alive
37	M	64	no	ccRCC	III	III	7×6×6	T3b	N1	M0	7.8	Dead
38	M	60	no	ccRCC	II	III	8×7×6	T2a	N0	M0	43.9	Dead
39	M	60	no	ccRCC	I	II	4×3.5×3.5	T1a	N0	M0	86.92	Alive
40	F	33	no	ccRCC	I	II	2.5×2.5×2	T1a	N0	M0	86.69	Alive
41	M	67	no	ccRCC	III	II	7×5.5×4	T3a	N0	M0	59.4	Dead
42	M	55	no	ccRCC	I	I	5×4×4	T1b	N0	M0	86.33	Alive
43	M	52	no	ccRCC	I	I	6.5×5.5×5.5	T1b	N0	M0	86.33	Alive
44	M	57	no	ccRCC	I	I	6×5.5×	T1b	N0	M0	86.33	Alive
45	M	52	no	ccRCC	I	I	5×4×3.5	T1b	N0	M0	86.30	Alive
46	M	60	no	ccRCC	III	IV	7×7×6	T3a	N0	M0	8.6	Dead
47	M	64	no	ccRCC	II	II	10×10×7	T2a	N0	M0	20.6	Dead
48	M	65	no	ccRCC	III	IV	5.5×5×4.5	T3a	N0	M0	9.5	Dead
49	F	75	no	ccRCC	I	I	5×4.5×4	T1b	N0	M0	84.69	Alive
50	F	61	no	ccRCC	III	IV	12×10×7	T3a	N0	M0	45.2	Dead
51	M	69	no	ccRCC	I	III	5×4.5×4.5	T1b	N0	M0	84.39	Alive
52	M	73	no	ccRCC	I	II	2.5×2.5×2	T1a	N0	M0	83.97	Alive
53	F	68	no	ccRCC	I	II	4.5×4.5×4	T1b	N0	M0	83.74	Alive
54	M	71	no	ccRCC	I	II	5×5×4.5	T1b	N0	M0	83.74	Alive
55	F	65	no	ccRCC	I	I	5×3.5×4	T1b	N0	M0	83.34	Alive
56	M	86	no	ccRCC	II	III	9.5×7×5.5	T2a	N0	M0	33.80	Dead
57	M	65	no	ccRCC	II	III	10×6×6	T2a	N0	M0	37.2	Dead
58	M	61	no	ccRCC	I	I	3.5×3×2.5	T1a	N0	M0	82.62	Alive
59	F	72	no	ccRCC	I	II	5×4.5×4	T1b	N0	M0	82.07	Alive
60	M	58	no	ccRCC	I	I	4.5×4×3.8	T1b	N0	M0	82.03	Alive
61	M	68	no	ccRCC	III	IV	6.5×6.5×5.5	T3a	N0	M0	11.3	Dead
62	M	74	no	ccRCC	II	III	8×8×5	T2a	N0	M0	81.25	Alive
63	M	38	no	ccRCC	III	IV	5.5×4.5×4	T3a	N0	M0	12.6	Dead
64	M	33	no	ccRCC	I	III	7×6×5	T1b	N0	M0	80.79	Alive
65	M	75	no	ccRCC	I	II	4×3×2.5	T1a	N0	M0	80.59	Alive
66	F	64	no	ccRCC	I	II	6.5×5.5×5	T1b	N0	M0	80.56	Alive
67	F	59	no	ccRCC	I	I	3×3×2.5	T1a	N0	M0	80.26	Alive
68	M	59	no	ccRCC	I	II	3.5×3.5×2.5	T1a	N0	M0	80.13	Alive
69	M	58	no	ccRCC	I	I	4.5×4.5×4	T1b	N0	M0	79.67	Alive
70	M	55	no	ccRCC	I	II	4×3×2.5	T1a	N0	M0	79.64	Alive
71	F	81	no	ccRCC	I	II	6×5.5×4.5	T1b	N0	M0	6.6	Dead
72	F	59	no	ccRCC	I	I	5.5×5×5	T1b	N0	M0	79.44	Alive
73	M	60	no	ccRCC	III	III	7×5×5	T3a	N0	M0	15.7	Dead
74	M	67	no	ccRCC	I	II	6×5×5	T1b	N0	M0	78.30	Alive
75	M	34	no	ccRCC	III	II	13×6.5×5.5	T3a	N0	M0	3.9	Dead
76	M	48	no	ccRCC	III	III	5.5×4.5×4	T3a	N0	M0	15.2	Dead
77	M	63	no	ccRCC	I	III	5×4×2	T1b	N0	M0	77.61	Alive
78	F	70	no	ccRCC	I	II	6×5×4	T1b	N0	M0	77.51	Alive
79	M	65	no	ccRCC	III	II	8×7×6	T3a	N0	M0	10.2	Dead

Table S4. siRNA sequence used in the study	
**siRNA**	**Sequence**
METTL14-1	S: GCAAAGATGAGCAGAGAGAAATTGCT
	AS: AGCAATTTCTCTCTGCTCATCTTTG
METTL14-2	S: GGGAGCTCATCAGGCTAAAGGATGA
	AS: TCATCCTTTAGCCTGATGAGCTCCC
ESRP2	S: AGCCCGAGGTGATAAAGCA
	AS: TGCTTTATCACCTCGGGCT
YTHDC1	S: CGACCAGAAGATTATGATA
	AS: GCTGGTCTTCTAATACTAT

Table S5. Primers used in RT-qPCR	
**Primers**	**Sequence**
METTL3	F: ATCCCCAAGGCTTCAACCAG
	R: GCGAGTGCCAGGAGATAGTC
METTL14	F: TCCCCATAATGATTACTGC
	R: CTGATGTCAAAGGCTTCTAT
WTAP	F: CTTCCCAAGAAGGTTCGATTGA
	R: TCAGACTCTCTTAGGCCAGTTAC
ALKBH5	F: GGCCGTATGCAGTGAGTGATT
	R: TGTCCGTGTCCTTCTTTAGCG
FTO	F: TCAGCAGTGGCAGCTGAAAT
	R: CTTGGATCCTCACCACGTCC
Lnc-LSG1	F: CTTGTGCCTCAGAATCATCATAGAC
	R: CCAGAAGGTGTTAATCATCATCACT
ESRP2	F: TGGTGTGGCCCTCTGTCTCAAC
	R: GCCCCCTGCAATCTTTACGAA
YTHDC1	F: CACCAGAGACCAGGGTATTTAAAGGATC
	R:CATTCCTTGCCAAGGTGGTGGTGGTCCCAT
GAPDH	F: GTGAAGCAGGCGTCGGA
	R: AGCCCCAGCGTCAAAGG

Table S6. The m6A level of lncRNAs downregulated in 786-O and Caki-1 shMETTL14-1 cells

lnc-SKIL-3	lnc-PHLDA1-1
OTUD6B-AS1	lnc-ANKRD55-6
lnc-IL17B-2	NCK1-DT
lnc-TMEM160-1	lnc-CARHSP1-1
lnc-ZFC3H1-10	lnc-SMARCC1-3
lnc-CA8-13	PCBP1-AS1
lnc-KLF10-2	lnc-TCFL5-6
lnc-UBE2Q1-2	lnc-FAM136A-1
lnc-FAM84B-20	lnc-SPOCD1-2
FGD5-AS1	lnc-EIF2AK4-6
lnc-EBF1-4	lnc-LBR-4
lnc-LGALS3BP-2	lnc-RFNG-2
lnc-PDE4D-7	lnc-SLC25A30-4
TUG1	lnc-TRIM37-1
lnc-PCDH15-2	lnc-JUNB-1
DDIT4-AS1	lnc-C1orf141-2
lnc-ZNF121-1	lnc-SLC25A32-4
lnc-GTF2A2-6	lnc-PFDN2-1
lnc-FAM92B-6	lnc-FSCN2-3
lnc-FOXS1-2	lnc-STAT1-2
VCAN-AS1	lnc-MRPL24-2
lnc-PRELID3B-3	lnc-SLC45A3-2
lnc-TAF12-5	lnc-PPP5D1-1
lnc-LSG1-4	NORAD
lnc-FAM166A-3	lnc-BAZ2A-2
lnc-MFSD8-8	lnc-RAB3IL1-2
NEAT1	lnc-CLMP-3
lnc-STPG4-3	lnc-RPL17-2
lnc-SLC43A3-1	lnc-NFS1-1
lnc-EMC1-1	lnc-ZBED5-4
lnc-MGLL-5	lnc-CA1-3
lnc-TMEM189-UBE2V1-4	lnc-TLDC1-2
lnc-CEP152-1	lnc-ARHGEF2-3
lnc-ZDHHC7-2	lnc-MAP4K2-3
lnc-CDC42BPB-4	MALAT1
lnc-RNF39-9	lnc-LTBP3-2
lnc-CCDC57-6	lnc-ISG15-5
lnc-ARID2-11	lnc-PADI2-1
lnc-EEF1E1-2	lnc-TSC1-1
lnc-CTSZ-7	lnc-S100A11-2
lnc-SHANK2-5	lnc-SAMD11-14
lnc-TXNDC16-3	lnc-PASK-2
PSMA3-AS1	lnc-C8orf89-3
lnc-ANKUB1-2	lnc-C9orf64-1
lnc-TFEC-5	lnc-MOS-1
lnc-TRPA1-2	lnc-PDGFB-4
lnc-ITGA11-6	lnc-SKAP2-6
lnc-DCAF1-1	lnc-ITGB1BP2-2
lnc-FRMD5-1	
lnc-SUMF1-18	
lnc-HNRNPU-1	
lnc-PUM2-3	
lnc-C5orf60-3	
lnc-TNPO2-1	
lnc-ENPP1-5	
LINC00958	
lnc-CALCRL-2	
lnc-SAMD11-13	
lnc-PAQR7-1	

Abbreviations list	
**Abbreviations**	**Full name**
ccRCC	Clear cell renal carcinoma
m6A	N6-methyladenosine
METTL14	methyltransferase 14
METTL3	methyltransferase 3
WTAP	WT1 associated protein
FTO	fat mass and obesity associated
ALKBH5	alkB homolog 5
TCGA	The Cancer Genome Atlas
CNVs	copy number variations
OS	overall survival
DFS	disease-free survival
lncRNA	long non-coding RNA
MeRIP-seq	methylated RNA immunoprecipitation sequencing
ESRP2	epithelial splicing regulatory protein 2
YTHDC1	YTH domain containing 1
IHC	immunohistochemistry
FISH	fluorescence in situ hybridization
HE	hematoxylin and eosin staining
CHX	cycloheximide
CQ	chloroquine
FGFR2	fibroblast growth factor receptor 2
